# Structural Insight into Host Recognition by Aggregative Adherence Fimbriae of Enteroaggregative *Escherichia coli*

**DOI:** 10.1371/journal.ppat.1004404

**Published:** 2014-09-18

**Authors:** Andrea A. Berry, Yi Yang, Natalia Pakharukova, James A. Garnett, Wei-chao Lee, Ernesto Cota, Jan Marchant, Saumendra Roy, Minna Tuittila, Bing Liu, Keith G. Inman, Fernando Ruiz-Perez, Inacio Mandomando, James P. Nataro, Anton V. Zavialov, Steve Matthews

**Affiliations:** 1 Center for Vaccine Development, Department of Pediatrics, University of Maryland School of Medicine, Baltimore, Maryland, United States of America; 2 Centre for Structural Biology, Department of Life Sciences, Imperial College London, South Kensington, London, United Kingdom; 3 Department of Chemistry, University of Turku, Turku, JBL, Arcanum, Turku, Finland; 4 Department of Chemistry and Biotechnology, Swedish University of Agricultural Sciences, Uppsala BioCentre, Uppsala, Sweden; 5 Paragon Bioservices, Inc, Baltimore, Maryland, United States of America; 6 Department of Pediatrics, University of Virginia School of Medicine, Charlottesville, Virginia, United States of America; University of Manchester, United Kingdom

## Abstract

Enteroaggregative *Escherichia coli* (EAEC) is a leading cause of acute and persistent diarrhea worldwide. A recently emerged Shiga-toxin-producing strain of EAEC resulted in significant mortality and morbidity due to progressive development of hemolytic-uremic syndrome. The attachment of EAEC to the human intestinal mucosa is mediated by aggregative adherence fimbria (AAF). Using X-ray crystallography and NMR structures, we present new atomic resolution insight into the structure of AAF variant I from the strain that caused the deadly outbreak in Germany in 2011, and AAF variant II from archetype strain 042, and propose a mechanism for AAF-mediated adhesion and biofilm formation. Our work shows that major subunits of AAF assemble into linear polymers by donor strand complementation where a single minor subunit is inserted at the tip of the polymer by accepting the donor strand from the terminal major subunit. Whereas the minor subunits of AAF have a distinct conserved structure, AAF major subunits display large structural differences, affecting the overall pilus architecture. These structures suggest a mechanism for AAF-mediated adhesion and biofilm formation. Binding experiments using wild type and mutant subunits (NMR and SPR) and bacteria (ELISA) revealed that despite the structural differences AAF recognize a common receptor, fibronectin, by employing clusters of basic residues at the junction between subunits in the pilus. We show that AAF-fibronectin attachment is based primarily on electrostatic interactions, a mechanism not reported previously for bacterial adhesion to biotic surfaces.

## Author Summary

Enteroaggregative *Escherichia coli* (EAEC) is a major cause of diarrhea worldwide and is commonly present as an infection in symptomatic travelers returning from developing countries. The attachment of EAEC to the human intestine is mediated protein filaments extending from the bacterial surface known as aggregative adherence fimbria (AAF). Here we use X-ray crystallography and nuclear magnetic resonance (NMR) structures to provide an atomic structure of the protein fibers made by the two major variants, AAF/I and AAF/II. The structures of the major subunit proteins show that the AAFs assemble into flexible, linear polymers that are capped by a single minor protein subunit at the tip. Biochemical assays reveal that the AAFs recognize a common receptor, the extracellular matrix protein fibronectin, via clusters of positively-charged amino acid residues running along the length of the fimbriae. Our structures suggest a unique mechanism based on ionic interactions for AAF-mediated receptor binding and biofilm formation.

## Introduction

Enteroaggregative *Escherichia coli* (EAEC) was first identified in 1987 and it has since become recognized as a leading cause of acute and persistent diarrhea worldwide [1], [2]. EAEC is characterized by its distinct “stacked-brick” pattern of aggregative adherence (AA) to HEp-2 cells *in vitro* [1]. This defining phenotype is mediated by aggregative adherence fimbriae (AAF). EAEC has been associated with persistent diarrhoea in children and in individuals infected with HIV [3], [4]. It is also commonly detected in symptomatic travelers returning from developing countries [5].

Emergence of a Shiga toxin (Stx)-producing strain of EAEC [6], [7] represents a significant threat to public health. The Stx-producing O104:H4 strain responsible for the 2011 outbreak in Germany was notable for being more virulent than Shiga-toxin–producing *E. coli* strains that do not have virulence factors associated with EAEC; it resulted in 3816 cases of gastroenteritis, 845 cases of hemolytic uremic syndrome (HUS), and 54 deaths [8].

Most enteric bacterial pathogens possess specific adherence factors that are responsible for recognizing receptors on host cells prior to colonization. In the case of EAEC, there are four known AAF variants: AAF/I (encoded by the *agg* gene cluster), AAF/II (encoded by the *aaf* genes), AAF/III (*agg3*) and Hda/AAF/IV (encoded by the *hda* genes) [9]–[12]. All AAF fimbrial biogenesis genes are encoded on a 55 to 65 MDa plasmid called pAA. Different AAF variants are expressed by different EAEC strains, where they are required for the bacterium's adherence to small and large intestinal mucosa. AAF adhesins have also been shown to promote biofilm formation on abiotic surfaces (glass and plastic) [13]. Despite their shared phenotypes, AAF show significant differences in agglutination of erythrocytes from different species [12], suggesting that they may recognize different receptors or bind to the same receptors with different affinity.

**Figure 1:**
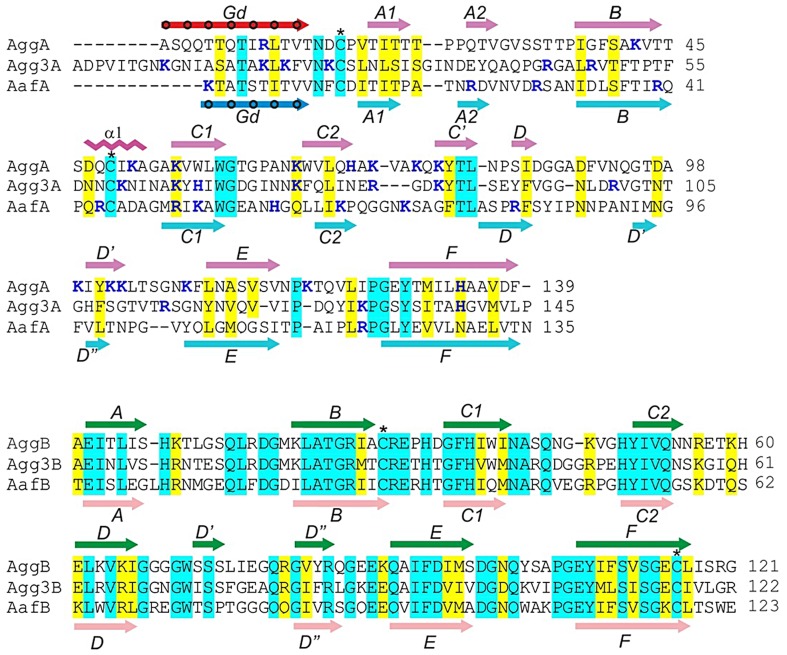
Sequence alignment of the major (AggA, AafA and Agg3A) and minor (AggB, AafB and Agg3B) subunits of aggregative adherence fimbriae (AAF) type I, II and III. Secondary structure elements of AggA, AafA, AggB, and AafB core structures are shown in magenta, cyan, green, and pink respectively, whilst the donor strands in AggA and AafA (Gd) are shown in red and blue, respectively. Donor residues occupying pockets of the acceptor cleft are indicated with circles. Amino acid identities and similar residues are indicated by background shading in cyan and yellow, respectively. The donor residues, occupying pockets of the acceptor cleft are indicated with circles. Positively charged residues are shown in bold and painted in blue. Cysteine residues involved in disulfide bonds are indicated with stars. CLUSTALW alignment of sequences was modified based on superposition of structures of the donor strand complemented (DSC) subunits AggA and AafA and AggB and AafB (this study).

AAF are assembled via the FGL chaperone/usher (CU) pathway [14], [15] and share a similar gene cluster architecture with those of the Afa/Dr subfamily of polyadhesins [16], [17]. As with Afa/Dr polyadhesins, AAF consist of two secreted protein subunits, a major subunit (A) and a putative minor subunit (B). The minor subunit of Afa/Dr polyadhesins may mediate invasion of host cells by uropathogenic *E. coli* [18]; usher-independent secretion has also been proposed [19], [20]. In EAEC, the minor subunit of AAF/II (AafB) has been associated with the release of cytokines [10], [21], suggesting its surface localization and function in EAEC pathogenesis. Whereas the minor subunits are relatively conserved within the subfamily, the major (A) subunits of AAF show only marginal sequence similarity among each other (Fig. 1) and no sequence similarity to the subunits from the Afa/Dr family (Fig. S1). Whereas most of the major subunits of CU-assembled fimbriae are typically negatively charged at physiological pH, the major subunits of AAF are positively charged up to pH 9.2–9.5; this feature is thought to play a role in EAEC adhesion. Moreover, a regulatory mechanism based on the repulsion of AAF and surface-localized dispersin has been proposed [22]. Although the major subunit of AAF/II has been recently shown to mediate attachment of EAEC strain 042 to the extracellular proteins fibronectin, laminin and type IV collagen [23], the mechanism of recognition is not known, and it is also not clear whether other types of AAF mediate attachment.

Previous transmission electron microscopy (EM) studies of the AAF expressed by *E. coli* showed the presence of thin, bundled fibers that can extend up to several microns from the bacterial surface. Estimates for the diameter of individual fimbriae have been reported be in the 2–3 nm range for AAF/I [9], [10], whereas images for AAF/II show fibers of up to 5 nm in width [10]. AAF/III are also long, flexible fimbriae with measured diameters of 3–5 nm, but these were usually observed as individual filaments and occasionally in bundles [24]. Based on homology of the CU machineries, it is be predicted that these AAF structures are assembled by the FGL CU pathway in a similar fashion to the *Yersinia pestis* F1 antigen [25], *E. coli* AFA-III [26], *E. coli* CS6 [27], *Yersinia pestis* pH6 antigen [28] and *Salmonella* Saf polyadhesins [29]. The major subunits from these systems lack the last (G) β-strand of a typical seven-stranded immunoglobulin fold, which exposes a substantial hydrophobic cleft running between the two β-sheets of the subunit. In the fiber, subunits are linked together by donor strand complementation (DSC) with an N-terminal G_d_ donor strand segment of one subunit inserted into the hydrophobic cleft of a neighboring subunit [25], [30].

Theoretically, for any fimbrial subunit capable of DSC polymerization it is possible to create a circularly permuted construct [26], [27], [31], [32]. In this construct, the Gd strand is placed at the C terminus of the subunit, enabling self-complementation and formation of a monomer with the classical Ig-fold. Whereas it is difficult to determine the structure of multimeric fimbriae at atomic resolution, production of stable monomers through donor strand complementation allows their study using nuclear magnetic resonance (NMR) spectroscopy and/or X-ray crystallography.

In this study, we report X-ray crystallographic and NMR studies of monomeric, donor-strand-complemented major and minor subunits of AAF/I from the German outbreak strain and AAF/II from EAEC strain 042 and provide an atomic resolution model for the structure of the entire fimbriae. Based on the structures and results of NMR titration experiments, site directed mutagenesis was performed to map the fibronectin binding sites in the organelles. Our results suggest that although there are significant structural differences between AAF/I and AAF/II, both uniquely rely on ionic-based mechanisms for adhesion to a common receptor.

## Results

### Model of AAF assembly and design of self-complemented subunits

Major subunits of AAF contain a pair of conserved cysteine residues that are commonly found in subunits of CU fimbriae (Fig. 1) [33], [34]. As the first cysteine often marks the beginning of the subunit core structure, we hypothesized that the N-terminal sequences 12–24 residues preceding the cysteine in the major subunits could act as donor strands connecting subunits in the fiber. At the same time, based on the fact that minor subunits of AAF are 12–23 residues shorter than major subunits (Fig. 1), we hypothesized that they do not possess their own donor strand sequences and are stabilized in the fiber by strands donated by major subunits. A model, in which minor subunits insert at the tip of a polymer of major subunits, would be consistent with this hypothesis.

**Figure 2:**
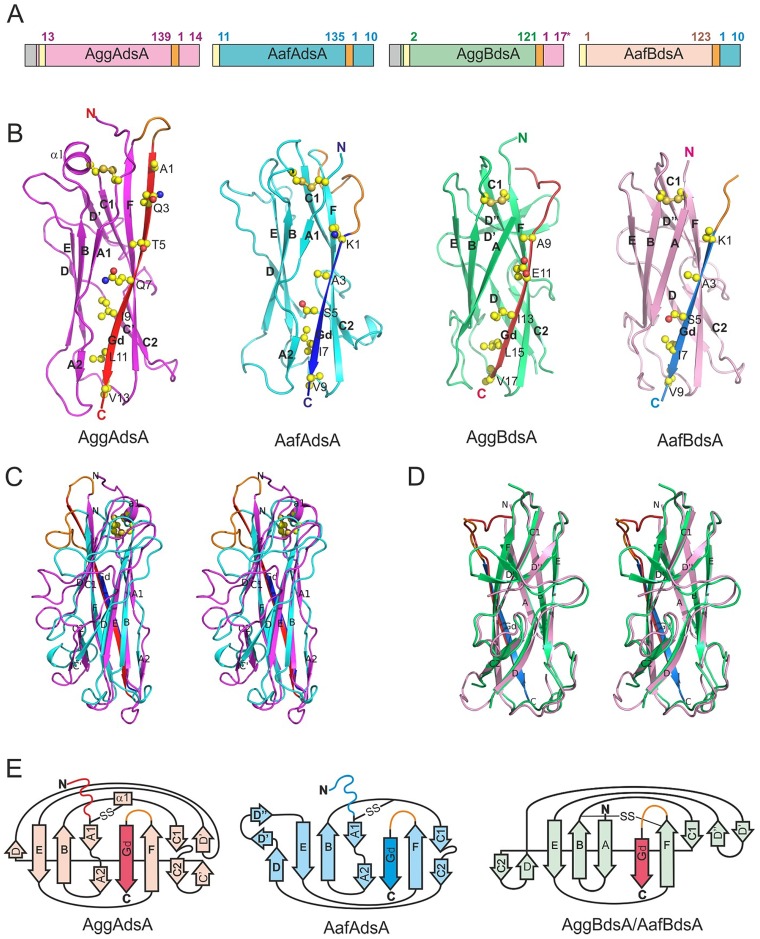
High-resolution structures of DSC subunits of AAF type I and II. (A) Schematic representation of DSC monomers. The positions of residues flanking mature sequence are numbered. Signal peptide, N-terminal His-tag, and linker are colored in grey, yellow and orange, respectively. For AggBdsA, both donor strand sequences of AggA from the German outbreak strain and classical EAEC strain 17–2 (three residue longer) were used successfully to stabilize AggB, however only the later crystallized. Amino acid sequences of the constructs are shown in Fig. S2. (B) Cartoon diagrams of AggAdsA, AafAdsA, AggBdsA and AafBdsA. (C and D) Structural superposition of AggAdsA and AafAdsA (C) and AggBdsA and AafBdsA (D) (stereo view). (E) Topology diagrams. The core structure of AggAdsA, AafAdsA, AggBdsA and AafBdsA is painted in magenta, cyan, green, and pink, respectively. The AggA and AafA Gd donor strands are colored in red and blue, respectively. The loop linking the core structure and donor strand (visible in AggAdsA, AafAdsA and AafBdsA) is shown in orange. Donor strand residues and disulfide bonds are shown as balls and sticks in A. Carbon, oxygen, nitrogen, and sulfur atoms are painted in yellow, red, blue and orange, respectively. Secondary structure elements are labeled in A and B.

To verify this model, we engineered DSC-monomers for each of the major A and minor B subunits of AAF type I and II, encoded by the *agg* and *aaf* gene clusters in the German outbreak strain and archetypal strain 042, respectively, by extending them with the potential donor strand sequences of the corresponding major subunits (dsA): AggAdsA, AggBdsA, AafAdsA and AafBdsA (Fig. 2A and S2). To ensure that the donor strand has sufficient conformational freedom to insert correctly into the acceptor cleft, we introduced linker sequences between the subunit's last residue and the first residue in the donor strand. Using native signal sequences, AggAdsA and AggBdsA were expressed in the *E. coli* periplasm, whereas AafAdsA and AafBdsA were expressed in the *E. coli* cytoplasm and refolded. All four subunits were purified in soluble, monomeric form and exhibited high stability, suggesting that the chosen donor strands sequences efficiently stabilized the subunits.

### High-resolution structures of AggAdsA, AggBdsA, AafAdsA and AafBdsA

To elucidate the structure and assembly mechanism of AAF, we determined high-resolution crystal structures of AggAdsA (1.6 Å; pdb accession code 4PH8), AggBdsA (2.4 Å; pdb accession code 4PHX) and AafBdsA (3.0 Å; pdb accession code 4OR1) as well as the solution structure of AafAdsA (pdb accession code 2MPV and BMRB ID 25001) (Fig. 2 and Fig. S3 and Tables S1 and S2).

The DSC-monomers of the major subunits (AggAdsA and AafAdsA) have a classical Ig-like fold that consists of two β sheets packed against each other in a β sandwich (Fig. 2B). In solution, the sequence prior to the first conserved cysteine (residues 1–10, Fig S2) is highly flexible (Fig. S3A), suggesting that the N-terminal extension would be fully accessible for polymerization in preassembly monomers. On the other hand, when the N-terminal extensions are linked to the C-termini of the subunits to form the missing C-terminal G strand and facilitate self-complementation, they are well ordered and illustrate how major subunits form DSC contacts in AAF fibers.

Superposition of the AggAdsA and AafAdsA structures revealed variation at the edges of the β sandwich structure (Fig. 2C and E). In AafAdsA, a substantial strand D is hydrogen bonded to strand E of β sheet 1 (A_1_BED) where as in AggAdsA, strand D is very short. Instead, it has additional strands C and D, which form the edge of β sheet 2 (CDC_1_C_2_FG_d_A_2_) in this subunit. Both strand F and the donor strand (G_d_) at the other edge of sheet 2 are longer in AggAdsA than in AafAdsA. The donor strand of AggAdsA inserts seven classical donor residues in to the acceptor cleft (Fig. 2B), which is two more than AafAdsA and other structurally characterized subunits from FGL CU systems. In addition, AggAdsA contains an α helix in the loop between B and C_1_ strands, which is absent in AafAdsA. These features give AggAdsA a more elongated shape than AafAdsA.

The overall similarity between AggA and AafA, which are the closest structural homologues, displays an r.m.s.d. of 3.2 Å for 125 Cα atoms. The AFA/III major subunit AfaE (pdb: 2ixq) [35] was identified as the second most structurally similar protein to AggA (r.m.s.d. of 4.1 Å for 120 Cα atoms) and AafA (r.m.s.d. of 4.0 for 115 Cα atoms) by a DALI [36] search of the protein data bank, confirming the evolutionary relationship between AAF and AFA/Dr fimbriae. The maximal structural differences between the AAF subunits and AfaE were found in the same structurally variable segments: the loop region between strands C_2_ and E, beginning of strand G_d_ and BC_1_ loop (Fig. S4). The most structurally conserved region in the three structures corresponds to the beginning of strand F and the ends of strands G_d_ and A_2_, which include conserved Gly127 and Tyr129 residues in strand F and Leu11 in the donor strand G_d_ (residues are numbered according to the sequence of AggA) (Fig. S1 and S5). Another conserved feature represents the disulfide bond connecting the BC_1_ loop (α helix in AggAdsA) and the beginning of the subunit fold. The donor strand in the AAF and AFA/Dr families shares the same interactions with its neighboring strand as in other FGL CU systems such as Saf [29] and the F1 antigen [25]. In these systems, the Gd strand lies at the sheet edge, whereas in the P-pilus and type 1 fimbriae it is shorter and sandwiched intimately between long A and F strands [37]. The lack of a second pairing strand for Gd is a distinguishing feature of the FGL CU systems that is reflected in the requirement for the long G’ strand in the chaperone [17].

The level of conservation of structural elements in major subunits generally correlates with the degree of their mobility in solution (Fig. S3). The conserved core structure of AafAdsA is considerably less mobile in the NMR ensemble than the structurally variable region including the sequence between strands D and E, the N-terminus, the BC_1_ loop, and the beginning of strand G_d_, all localized at the upper part of the molecule (Fig. S3B). To provide further characterization of mobility in these regions, heteronuclear NOE and T_2_ relaxation data were recorded and analyzed. These data together with TALOS+ predictions [38] confirmed that the region between residues 89 to 96 is dynamic on the picosecond timescale. Not surprisingly, the engineered linker between the C-terminus and the G_d_ strand is also highly flexible.

**Figure 3:**
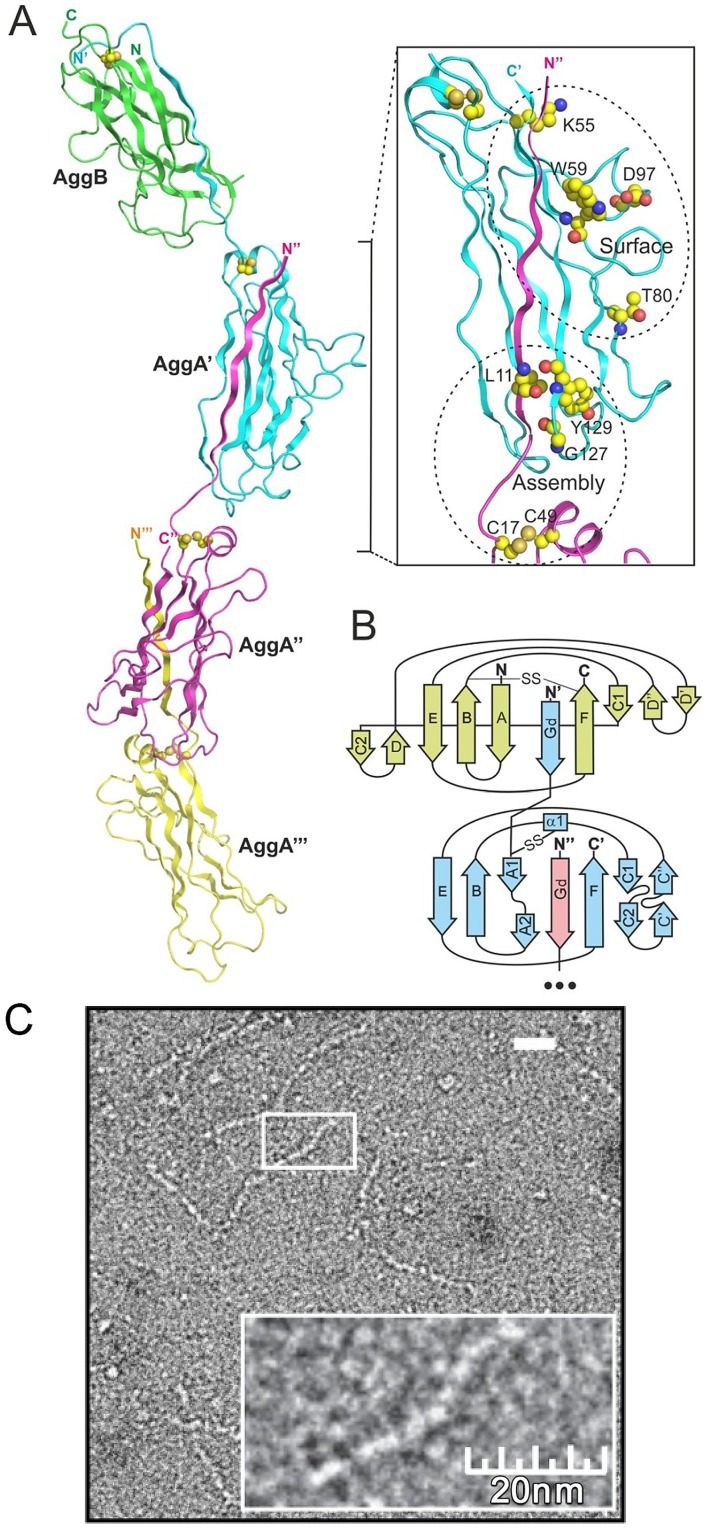
AAF architecture. (A) Model of AAF/I constructed based on the crystal structures of DSC subunits, AggAdsA and AggBdsA, and the crystal structure of the F1 antigen mini-fiber [25] (cartoon diagram). The fiber contains a single copy of the AggB subunit (green) at the tip of a polymer of the AggA subunits (a fragment containing three AggA subunits is shown). The insert shows localization of conserved residues in the structure of the fiber. (B) Topology diagram of the AAF/I fiber. (C) Negative stain transmission electron micrographs of diluted AAF/II fimbriae isolated from enteroaggregative *E. coli* strain 042.

Interestingly, many residues that are either conserved across both AAF/AFA families (Fig. S1) or conserved only within AAF major subunits (shaded blue in Fig. 1) are located in the structurally variable and flexible regions (Fig. S5). Specifically, four of these residues are highly surface exposed, namely the AAF specific basic residue position 55, Trp59, Thr80 and the AAF/AFA conserved asparagine/aspartate 97 (residues are numbered according to the sequence of AggA), (Fig. 3A insert, Fig. S5).

The minor subunits AggBdsA and AafBdsA have more similar structures than the major pilins: 117 matching Cα atoms are superimposed with r.m.s.d. of 1.2 Å (Fig. 2B and D). The donor sequences derived from the corresponding major subunits form classical donor strands (G_d_), each inserting five donor residues into the subunit core structure. The values of shape correlation statistic (*S_c_*) [39] calculated for the acceptor cleft-donor strand interactions for AggBdsA and AafBdsA (0.733 and 0.756, respectively) were similar to those calculated for AggAdsA and AafAdsA (0.784 and 0.804, respectively). The good geometrical fit between the donor strands and acceptor clefts suggests that these interactions are native [27]. As predicted, the minor subunits have no N-terminal extension that could act as a donor strand: the first residue in the mature protein is structured and immediately followed by the first strand A. Hence, this structural evidence suggests that the minor subunits assemble by accepting donor sequences of the corresponding major subunits. As such sequences are only available in tip subunits of major subunit polymers, the AAF minor subunits are likely to be tip localized. This arrangement is consistent with EM localization studies of the AAF pilins. Bacteria lacking the major subunit are unable to assemble fimbrial surface structures and immunogold localization revealed the major subunit distributed along the fimbrial shaft [10]. Furthermore, the significant sequence identity with the AfaD tip invasin (>60%),which has been visualized as a tip subunit, together with the ability of AggB to functionally complement AfaD mutants are also suggestive of a similar localization for AAF minor sununits [35], [40], [41].

The topology of minor subunits differs from that of the major subunits in several other aspects (Fig. 2E). In minor subunits, strand A is not split between the β sheets, but locates entirely in the sheet 1. Instead, strand C_2_ that in a classical Ig fold is hydrogen bonded to strand F, in minor subunits is hydrogen bonded to strand D and hence belongs to sheet 2. Strands D and D ´ in sheet 2 of minor subunits have no analogues in the structure of major subunits. This region is the most dissimilar (Fig. 2C) and flexible (Fig. S3) in the structure. A conserved disulfide bond also stabilizes the minor subunits. However, this bond connects the BC_1_ loop with the end of strand F and not the beginning of the fold as in major subunits. The distinct structure, high structural conservation and specific localization suggest a highly specialized important function for the AAF minor subunits.

### Atomic model of AAF

The structures of DSC monomers were used to model AAF (Fig. 3 and S6). The modules of the AAF shaft structures (-subunit:G_d_-) were modeled based on AggAdsA or AafAdsA, while the corresponding terminal modules (subunit:G_d_-) were modeled based on AggBdsA or AafBdsA. To revert the circular permutation of the DSC-monomers, the artificial linker sequences connecting C-termini of the DSC subunits with N-termini of donor strands were deleted and the C-termini of donor strands were bridged with N-termini of adjacent DSC subunits using native linker sequences TND and VNK in AggA and AafA, respectively. The fiber model was generated by assuming the same orientation between successive subunits as observed in the crystal structure of the mini-fiber of the F1 antigen [25].

To provide further evidence for these models we visualized AAF/II fimbriae purified from the prototypical enteroaggregative *E. coli* strain 042 using negative-stain transmission electron microscopy. Consistent with previous EM studies of bacterial-surface localized AAF fimbriae [10], [11], our purified AAF fibers show a propensity to intertwine into bundles at high concentrations. However, upon dilution individual fimbriae can be clearly observed (Fig. 3C). These fibers are thin with diameter of 2.5–4 nm and highly flexible, occasionally exhibiting ∼30° bends within relatively short stretches (20 nm, ∼5 subunits). The extended appearance of these fibrillar structures and their inherent flexibility are consistent with the relatively small interdomain contact area present in our reconstructed models. Similar low resolution EM images have been obtained for the FGL-CU Saf fimbriae and subsequent reconstruction revealed thin, extended structures of ∼2.5 nm width [42]. The dimensions of the observed fibers are also consistent with high-resolution structures for other FGL-CU organelles, namely the Caf1M-Caf1’-Caf1” mini-fiber of the F1 antigen[25] and the AfaDE tip complex [35], which show very similar values for the intersubunit angle (Fig. S6).

The more elongated AggA produced a longer AAF/I fiber than that of AAF/II consisting of the same amount of shorter AafA subunits (Fig. S6). The edge of the fiber module that is involved in DSC (strands A and G_d_) forms the less exposed side of the fiber helix. This region contains the majority of the conserved structural residues that are closely positioned and involved in assembly. The disulfide bond stabilizing the donor strand linker in major subunits functionally belongs to this region. The disulfide bond in minor subunits serves a different function in that it stabilizes the acceptor cleft (Fig. S7). The opposite segment of the structure (sequences between strands B and E) is more exposed. This mobile and structurally variable, yet conserved surface region is a potential candidate for a receptor binding site (Fig. 3A, insert).

### Fibronectin is a common receptor for AAF/I and AAF/II

EAEC adheres to fibronectin, and in strain 042 the attachment is mediated by AAF/II [23]. To examine if fibronectin is a common receptor for AAF, we studied AggAdsA-fibronectin interactions using surface plasmon resonance (SPR) (Fig. S8). The experiment revealed specific binding with a dissociation constant of 16±2 µM (Table 1). This value is similar to that previously found for AafAdsA [23]. Interestingly, AggBdsA also bound to fibronectin but with significantly lower affinity (∼100 µM). Whereas a single AggB is unlikely to promote attachment of bacteria to fibronectin, a polymer of AggA subunits would mediate a tight bacterial adhesion by establishing multipoint interactions with several fibronectin molecules.

**Table 1:** Effects of ionic strength and amino acid substitutions on the dissociation constant for AggAdsA-fibronectin binding.

Mutation	K_D_, μM
WT, 75 mM NaCl	16±2
WT, 300 mM NaCl	>200
Trp57	31±6
Trp59	17±3
Phe91	20±3
Arg10	23±3
Lys51 and Lys109	49±4
Lys55 and Lys103	56±5
Lys73 and Lys76	61±6
Lys73, Lys76, and Lys78	>200

### AAF-fibronectin binding is mediated by ionic interactions

**Figure 4:**
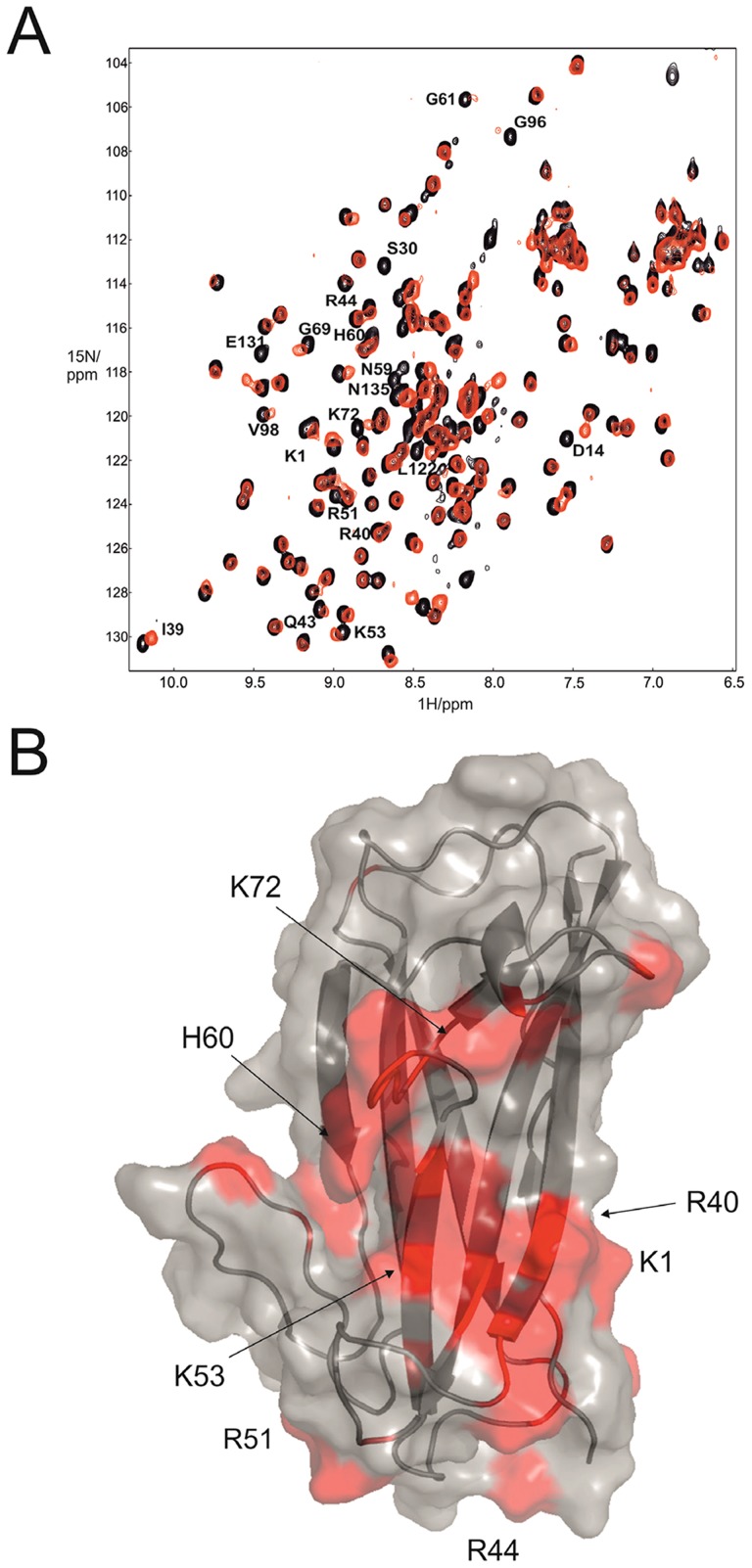
Fibronectin binding site mapping using NMR. (A) Overlaid ^1^H-^15^N HSQC NMR spectra for free AafAdsA (black) with two molar equivalents of fibronectin (red). Residue labels represent mature protein sequence numbering. (B) Map of chemical shift perturbations on the structure of subunit. Key basic residues are highlighted.

**Figure 5:**
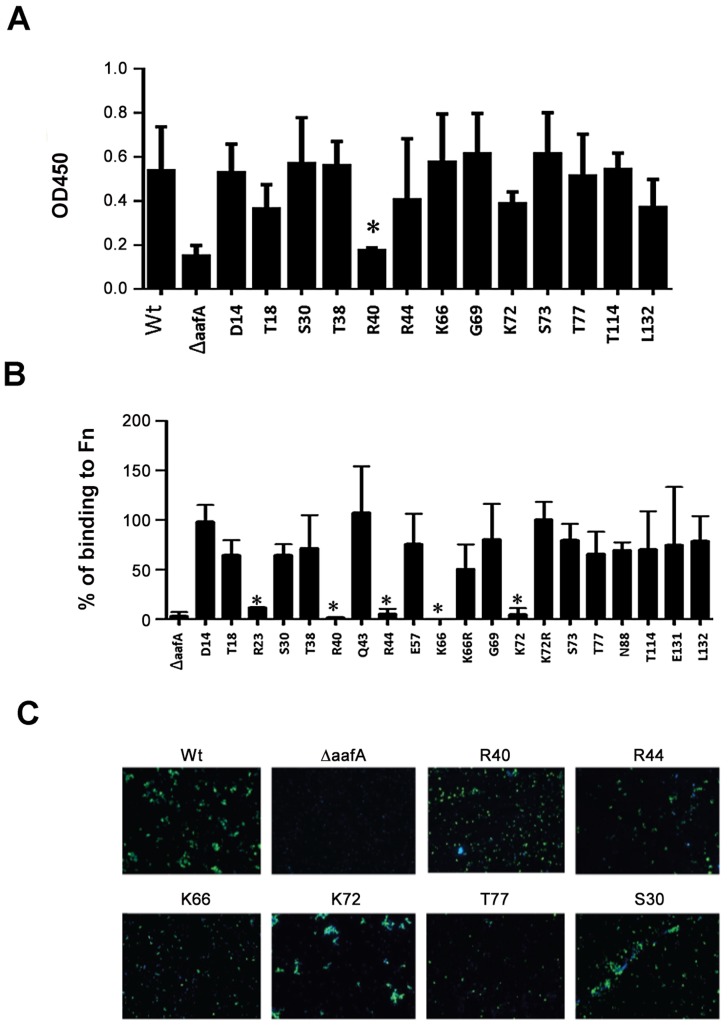
Involvement of AafA and site-directed-mutants in biofilm formation and binding to fibronectin. (A) Biofilm quantification. Bacteria were cultivated in LB for 20 h at 37°C in 24-well dishes and induced with 0.2% arabinose. Biofilms were fixed and stained with crystal violet, and then the stains were solubilized and quantitated spectrophotometrically at 450 nm. The bars represent the means of the results from triplicate wells; error bars indicate one standard deviation. Wt, wild type EAEC042, ΔaafA, EAEC042*aafA* mutant. All residues tested were mutated to Ala except Thr18Ile and Thr38Leu. *, P<0.005. (B) Fibronectin binding. EAEC 042 derivatives harboring site mutations in *aafA* were added to 96-well plates coated separately with 25 µg/ml of fibronectin, and the binding was determined by collecting the cells adhered to wells by scraping them into PBS. The number of adherent bacteria was determined by counting the resulting colonies in duplicate and normalized to the wild type adherence (as a 100% adherence). The bars represent the means for three experiments, with the error bars indicating 1 standard deviation. *, significantly different from EAEC042WT (*P*<0.005).(C) AafA surface expression. After induction of bacterial strains with 0.2% arabinose, cells were harvested, washed twice with PBS, and incubated with a polyclonal anti-aafA antibody (2 µg/ml) in PBS plus 1% bovine serum albumin for 1 h at room temperature with agitation. They were washed twice with PBS and incubated with a goat anti-rabbit IgG-fluorescein isothiocyanate (FITC) conjugate (10 µg/ml) for 30 min at room temperature in the dark. The washings were repeated, and the samples were resuspended in DAPI (4 mg/liter) and spread on slides for viewing by fluorescence microscopy.

An NMR titration experiment was performed to identify the putative fibronectin-binding interface in AafAdsA. After comparing ^1^H-^15^N HSQC NMR spectra of AafAdsA recorded in the absence and presence of FnI (Fig. 4A), several residues could be identified that exhibited significant chemical shift perturbations, suggesting the likely location of the binding surface (Fig. 4B). Unsurprisingly given the basic nature of AafA, a number of positive charged residues experienced chemical shift perturbations, namely Lys1, Arg40, Arg44, Arg51 and Lys53 and Lys72. Key residues in AafAdsA identified in the NMR FnI titration analyses were targeted for mutagenesis on the native fimbrial structure expressed in *E. coli*. Site-directed mutations were created in plasmid pBAD30 harboring the intact native *aafA* gene. Mutants were introduced into the 042Δ*aaf* strain, and fibronectin binding in the complemented mutants was scored in the presence of arabinose (to induce *aafA* expression). A number of additional residues (Thr18, Arg23, Thr38, Lys66 and Thr114) were chosen, which are located in approximately equivalent regions to reported protein-binding interfaces for AfaE, namely carcinoembryonic antigen (CEA) and DAF [26], [43], [44]. Although AafA is not reported to bind to either CEA or DAF, it is conceivable that these surfaces on AafA are also important for fibronectin binding and/or biofilm formation. Protein expression was confirmed by Western blot (Fig. S9) and, perhaps with the exception of T18I, AAF production in the mutant strains is similar to wild-type. These expression data also imply that mutant subunits are stably polymerized into fimbriae. This is also reflected in surface exposure levels assessed qualitatively using immunofluorescence microscopy of AafA mutants Ser30Ala, Arg40Ala, Arg44Ala, Lys66Ala, Lys72Ala, and Thr77Ala (Fig. 5C). It, however, cannot be ruled out that some variation in AAF presentation may contribute to the fibronectin binding or biofilm phenotypes.

AafA mutant Arg40Ala exhibited significant reduction of biofilm formation (Fig. 5A), while mutants Arg23Ala, Arg40Ala, Arg44Ala, Lys66Ala, and Lys72Ala displayed reductions in fibronectin binding compared with wild type (Fig. 5B). All other mutations did not affect either biofilm formation or Fn binding, including conservative substitutions Lys66Arg and Lys72Arg, which did not affect Fn binding. These data confirmed the identity of several residues suggested by NMR spectroscopy, and implicated two regions at the poles of the AafA subunit. Our data also suggested that the predicted receptor-binding site for DAF in the Dr adhesins is not involved in Fn binding. Interestingly, all residues identified as functional in fibronectin binding were basic arginines or lysines. As the high content of basic residues is a prominent feature for AAF, we hypothesized that basic residues are important for the AAF/I-fibronectin interactions. As EM studies of both bacterially-displayed and sheered AAF revealed an abundance of bundled fibers, it is also possible that there is a significant electrostatic contribution to this morphology and these intertwined bundles are important for function. This is consistent with the observation of a strong dependence of the strength of AggAdsA-fibronectin binding on the ionic strength of the solution (Table 1, Fig. S8). The affinity dropped by more than 12 fold in presence of 300 mM NaCl in 50 mM Hepes buffer, which could be explained by disruption of salt bridges between the interacting molecules.

To verify this hypothesis, we reduced the charge on the AggA surfaces that correspond to the equivalent basic regions identified in AafA by mutating groups of positively charge amino acids to alanine and measuring the interaction of the self-complemented monomer (i.e. AggAdsA) with fibronectin using SPR (Table 1). We focused the mutagenesis on surface patches comprising proximal pairs of lysines (including Lys51 and Lys109, Lys55 and Lys103, and Lys73 and Lys76) and the three closely positioned lysines Lys73, Lys76, and Lys78. All the double mutants exhibited significant decreases in fibronectin binding and, despite the absence of the cooperatively expected for a polymeric AggA fiber, the affinity for the triple mutant of monomeric AggAdsA dropped below the detection limit of the experiment. To confirm the structural integrity and stability of these charge removal mutations, the AggAdsA triple mutant (Lys73Ala, Lys76Ala, and Lys78Ala) was characterized further by circular dichroism (CD). The CD spectra of both WT and mutant monomeric proteins are almost identical showing a negative band at 217 nm and positive band at 195 nm (Fig. S10A). The CD profiles are characteristic of a highly similar β-sheet structure and thermal denaturation revealed that the mutations do not affect stability of the monomeric AggAdsA subunit (Fig. S10B). The defect in fibronectin binding for the mutant is therefore due exclusively to the absence of specific positively charged side chains. The low affinity and electrostatic nature of this interaction raises the question that AAF may target acidic proteins nonspecifically. We examined this using SPR and show that this is unlikely, as binding of AggAdsA to fibronectin is unaffected by the presence of 6-fold molar excess of Bovine serum albumin (BSA pI = 4.7; Fig. S11).

The conserved surface tryptophan residue in AAF major subunits (Trp59 and Trp55 in AggA and AafA, respectively) is located close to the conserved positive charges implicated in the AggA-fibronectin binding (Fig. 3A). In addition, AggA contains a closely positioned surface Trp57 as well as the exposed Phe91 and partially exposed Ile85, located in the variable region. To study the possible involvement of the extensive hydrophobic surface of these residues in AggA-fibronectin binding, we mutated each residue to produce the Trp57Ala, Trp59Ala, Ile85Ala and Phe91Ala mutants of the monomeric AggAdsA subunit. Trp57Ala, Trp59Ala, and Phe91Ala, but not Ile85Ala, were expressed, demonstrating that they are not essential for the protein structure. Examination of the fibronectin-binding of these AggAdsA mutants with the SPR binding assay revealed affinities similar to that of the wild type subunit (Table 1), suggesting that the large hydrophobic surfaces displayed on AAF subunits are not involved in the fibronectin binding. Furthermore, the prominence of basic lysine and arginine residues suggests that fibronectin binding by AAF/I and AAF/II is driven by electrostatic interactions.

### Modeling of fibronectin binding sites on AAF

**Figure 6:**
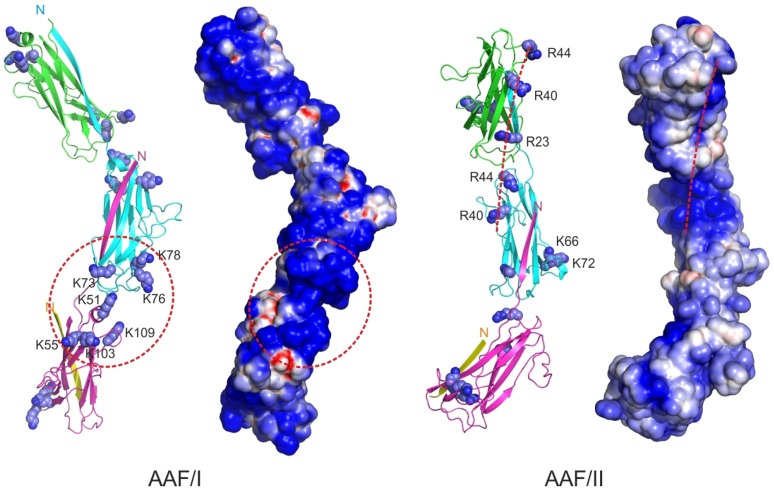
Location of fibronectin binding sites in AAF type I and II. Cartoon diagrams (left) and electrostatic potential assessable surfaces (right) of three major subunit polymers are shown. Positively charged residues interacting with fibronectin are drawn as sphere models. Arg40 in AAF/II is also involved in biofilm formation. Regions of positive surface potential are depicted in blue and negative potential in red (scale is from −4 to 4 kT/e). AAF/II contains two fibronectin binding surfaces: a continuous array of arginines 23, 40, and 44 and a repeated patch of lysines 66 and 72. In AAF/I, the basic residues concentrated at the interface between adjacent subunits. Seven lysine residues 51, 55, 73, 76, 78, 103 and 109 in this region are involved in fibronectin binding.

Superimposition of fibronectin-binding residues on the fiber models placed the experimentally determined fibronectin-binding pocket within the clefts formed by adjacent subunits in the fiber (Fig. 6). The pocket is particularly well pronounced in AAF/I. Three closely positioned lysines (73, 76 and 78), mutations of which practically abolished the binding, are located in the loop between the C_2_ and Ć strands at the bottom of the subunit. The other binding residues are located close to the top of the molecule: lysines 51 and 55 in the helix within the BC_1_ loop and at the beginning of strand C_1_, respectively, and lysines 103 and 109 at the end of strand Ć ´ and in the Ć ´E loop, respectively. These two polar segments are closely positioned in the fiber and form a continuous surface, which is characterized with the highest positive potential and contains a shallow groove in the middle. Similarly, in AAF/II all implicated binding residues locate at the junction between subunits with the fiber. Three arginines; 23 (end of the strand A_2_) at the bottom of AafA; 40 (beginning of strand B); and 44 (the BC_1_ loop) at the top of AafA are a part of the AAF/II surface with the highest positive potential. Interestingly, arginines 23, 40, and 44 form an array along the fiber length. Lysines 66 and 72 form an independent cluster locating at the subunit interface. It is likely that these residues contribute to fibronectin binding by forming additional contacts, which likely result from changes in the range of relative domain orientations along the fiber due to its flexibility. Alternatively, they might contribute to binding indirectly, either by promoting fiber bundling or stabilizing a binding competent conformation of the whole fiber. Although the majority of the identified binding residues are not conserved at specific sequence position (Fig. 1), many of them locate in the structurally similar regions at the poles of the subunits, which in the context of AAF fibers positions a high concentration of basic residues in the cleft between subunits.

## Discussion

The AAF of EAEC is a multifunctional organelle that contributes multiple phenotypes plausibly related to pathogenesis. AAF have been associated with adherence to human intestinal explants [45], biofilm formation on abiotic surfaces [13] and adherence to polarized T84 cell monolayers with stimulation of IL-8 release and opening of epithelial tight junctions [46]. Although no single receptor has yet been associated directly with these phenotypes, binding to extracellular matrix proteins has been associated with colonization and biofilm formation. The AAF adhesins can be classified into at least four variants [12] with conserved characteristics, despite low levels of amino acid identity among the major pilin subunits. In this work, we present the structures of the major and minor pilin subunits of the most common AAF variants, AAF/I and AAF/II. We find that despite less than 25% amino acid identity between the AAF/I and AAF/II major pilins, AggA and AafA respectively, the two pilin proteins adopt a similar fold with shared surface characteristics. The most notable feature of the AggA and AafA structures is the unusual surface distribution of basic residues, giving rise to subunits with a substantial net positive charge at physiologic pH. Although the majority of bacterial adhesins are not highly positively charged, the adhesin of *Stenotrophomonas maltophilia* is basic and has also been associated with biofilm formation and adherence to indwelling vascular catheters [47]. The contribution of positively charged adhesins to pathogenesis for either *Stenotrophomonas* or EAEC has largely been overlooked.

We present a new structural insight into the architecture of AAF/I and AAF/II fimbriae and reveal a contiguous basic surface located at the subunit interfaces within the biopolymer. Although the observation of highly basic AAFs in bundles is somewhat surprising due to potential repulsion between entwined fimbriae, intersubunit repulsion between subunits within a fiber may enable these long, thin fimbriae to project from the bacteria surface as far as possible and prevent them from collapsing onto it. The alignment of AAFs into bundles may be a consequence their extended length and inherent helicity. There is precedent in other biological fibers from bacteria; for example, in the Type IV pilus from *N. gonorrhoeae* extensive phosphorylation does not abrogate bundle formation, but reduces the curliness of fibers and thickness of bundles [48]. Furthermore, counterion coating of highly-charged surface of nanometer fibers has been shown to promote bundle formation [49].

We show that the basic nature in AggA and AafA plays an important role in the specific recognition of fibronectin. The mode of recognition is principally by electrostatic interaction, which is in contrast to mechanisms utilised by other bacterial fibronectin-binding proteins. Perhaps the best characterised structurally are the MSCRAMMs (microbial surface components recognizing adhesive matrix molecules) from Gram positive bacteria. MSCRAMMs are large modular proteins covalently attached to the bacterial cell wall. Examples of this family from staphylococci and streptococci bind fibronectin with high affinity through interaction with an extensive region with several copies of a repeat sequence. Structural studies have revealed that the repeats are disordered in isolation, but in complex they augment the β-sheets of fibronectin type I domains, forming an extensive β-zipper along the edge of consecutive domains, resulting in an extremely tight interaction with nM dissociation constants [50]. While the dissociation constants for single pilins lie in the μM range, the high avidity from the polymeric architecture of AAF would equate to a much stronger interaction with fibronectin. Although the precise role of fibronectin binding in EAEC pathogenesis is unclear, the strength of interaction and conserved characteristics between AggA and AafA fibronectin binding suggest a significant involvement. This also consistent with the persistent nature of EAEC infection which implies that bacteria progress beyond simple apical attachment on the epithelial surface to maintain disease. Indeed, while extracellular matrix proteins are usually localized to the remote basement membrane, access to a bacterial pathogen can occur during inflammation, invasion or specific breaching of tight junctions. Tight junction opening has been associated with AAF expression during the EAEC infection of T84 cells [46]. This may be the consequence of a colonization signal cascade in which proinflammatory cytokines are secreted from infected epithelial cells [21]. The receptor associated with this phenotype has yet to be identified, however we expect that our structural insight into the electrostatically driven mechanism for AAF/II binding provides new inspiration for mutagenesis experiments that may accelerate this effort. It is also plausible that a natural receptor for AAF adhesins is not fibronectin itself, but another or multiple extracellular protein(s). In addition to fibronectin, other host receptors have been implicated in EAEC adhesion [51] and more recently both laminin and cytokeratin8 (CK8) have been confirmed to interact with the major subunit of AAF/II fimbriae [52]. It is conceivable that AAF have evolved a more generalised electrostatic mechanism for binding several host receptors that have appropriate arrangements of acidic residues, negatively-charged glycosylation sugars and/or phosphorylated site.

We have also determined the structures of the minor pilin subunits AggB and AafB. As expected from the similar major pilin subunit structures and a higher degree of sequence conservation between the minor pilin subunits, the structures of the two minor pilin proteins are much more conserved. Although the role of the minor pilins in AAF is unknown, it has been suggested [17], and our data support this model, that the minor subunit caps the end of the AAF structure. Bacterial fimbriae are in general highly immunogenic, and antibodies raised against the structures are characteristically protective against bacterial colonization [53]–[55]. As such, fimbriae are high-profile vaccine candidates, and structural information can advance vaccine development efforts dramatically [56].

## Materials and Methods

### Construction of donor strand complemented monomers

The AggAdsA and AafAdsA expression constructs (pET101D-AggAdsA-O104H4 and pQE30-AafAdsA, respectively) were created as described previously in [57] and [58], respectively. The sequence coding for the *aggBdsA* gene was ordered from GenScript and placed under T7 promoter of pET101D to create the expression plasmid pET101D-AggBdsA. The sequence coding for the *aafBdsA* gene was ordered from Invitrogen and placed under T7 promoter of pQE20 to create the expression plasmid pQE30-AafBdsA.

### Protein preparation

The *aafAdsA* and *aafBdsA* constructs were transformed into *E. coli* strain M15 containing the pREP4 plasmid (Qiagen). Cells were grown at 37 °C in either LB or M9 minimal medium supplemented with ^15^NH_4_Cl and/or ^13^C-glucose (Cambridge Isotope Laboratories) and expression induced with 1 mM isopropyl β-D-1-thiogalactopyranoside (IPTG) at an OD_600nm_ = 0.6. Cells were harvested after 4 hrs, resuspended in 50 mM sodium phosphate pH 8.0, 8M urea, 300 mM NaCl, lysed using a French press, before being purified under denaturing conditions with Ni–NTA. The eluates were first dialysed against 50 mM sodium acetate pH 5, 50 mM NaCl, 1 M urea, followed by a second dialysis against the same buffer with no urea and finally gel filtered using a Superdex 75 gel-filtration column (GE Healthcare).

AggAdsA was expressed in the periplasmic space of *E. coli* strain BL21-AI, extracted by osmotic shock and purified as described in [57]. AggBdsA was expressed and extracted using the same procedure [57]. To remove the majority of contaminating proteins, the extract was filtrated though a 20-ml Source 30Q column (GE healthcare) in 20 mM Tris-HCl, pH 8.5. The sample was dialyzed overnight in 20 mM HEPES, pH 7.2 buffer, and purified further by cation-exchange chromatography on a Mono-S 5/50 GL column (GE healthcare) using a 0–250 mM elution gradient of NaCl. To obtain highest purity samples, protein was subjected to gel-filtration on a Superdex 75 column (GE Healthcare) equilibrated with 50 mM HEPES, pH 7.5 and 150 mM NaCl. Protein was concentrated to 33 mg ml^−1^ for crystallization experiments on a Vivaspin device (GE healthcare) with molecular weight cut-off of 5 kDa.

### Crystal structure determination

Crystallization of AafBdsA was performed by “sitting-drop” vapor-diffusion method grown in 0.1 M Bis-Tris pH 5.5, lithium sulphate and 25% w/v PEG 3350. Crystals were soaked for 30–60 s in cryoprotection solution (well solution complimented with 20% PEG 400) and then cooled by plunging them into liquid nitrogen. Diffraction data were collected under liquid-nitrogen cryoconditions at 100K on beamline I24 at the Diamond Light Source (DLS), UK. Data were processed with XDS [59]. The protein structure was solved by molecular replacement method using DraD protein (pdb accession code 2AXW: [60]) as a model using *Phaser* [61] and refined with Refmac [62]. Coordinates have been deposited with the protein databank with accession code 4OR1.

Crystallisation and the quality of preliminary diffraction data for AggAdsA have been described previously [57]. Crystallization of AggBdsA was performed by “sitting-drop” vapor-diffusion method using commercial screening kits Index-HR2-144, JCSG+ Suite (Qiagen) at 290 K. Crystals grew in drops with 0.2 M Lithium sulfate monohydrate, 0.1 M Tris HCl pH 8.5, 30% w/v PEG 4000. Crystals were soaked for 30–60 s in cryoprotection solution prepared by mixing two parts of precipitant solution with one part 50% PEG 400 and then cooled by plunging them into liquid nitrogen. Diffraction data were collected under liquid-nitrogen cryoconditions at 100K on beamline ID23-1 at the European Synchrotron Radiation facility (ESRF), Grenoble, France. Data were processed with XDS [59]. The protein structure was solved by molecular replacement method using DraD protein as a model (55% of sequence identity) using *Phaser* from the *PHENIX* Software package [61].

### NMR structure determination

For AafA-dsA, backbone and side-chain assignments were completed using our in-house, semi-automated assignment algorithms and standard triple-resonance assignment methodology [63]. H_α_ and H_β_ assignments were obtained using HBHA (CBCACO)NH. The side-chain assignments were completed using HCCH-total correlation (TOCSY) spectroscopy and (H)CC(CO)NH TOCSY. Three-dimensional ^1^H-^15^N/^13^C NOESY-HSQC (mixing time 100 ms at 800 MHz) experiments provided the distance restraints used in the final structure calculation. The ARIA protocol [64] was used for completion of the NOE assignment and structure calculation. The frequency window tolerance for assigning NOEs was ±0.04 ppm and ±0.06 ppm for direct and indirect proton dimensions and ±0.6 ppm for both nitrogen and carbon dimensions. The ARIA parameters p, Tv and Nv were set to default values. 144 dihedral angle restraints derived from TALOS were also implemented [65]. The 10 lowest energy structures had no NOE violations greater than 0.5 Å and dihedral angle violations greater than 5°. Although structure calculations readily converged without the introduction of manual assignments, a systematic check of automatically-assigned NOEs was carried out. The structural statistics are presented in Table S1.

### NMR titrations

^1^H-^15^N TROSY/HSQC spectra of ^15^N-AafA-*dsc* was recorded at 25µM in the absence and presence of the 30-kDa N-terminal type I repeat domain of fibronectin (FnI) in equimolar concentrations.

### Construction of AafA and AggA mutants

Bacterial strains were grown in Luria-Bertani (LB) broth at 37°C with shaking unless otherwise indicated. When appropriate, the medium was supplemented with antibiotics at the following concentrations: ampicillin, 100 µg/ml and kanamycin 50 µg/ml. The bacterial strains used in this study were DH5α [supE44 ΔlacU169 (φ80 lacZΔM15) hsdR17 recA1 endA1 gyrA96 thi-1 relA1], EAEC042WT, and EAEC042*aafA* [10].

### Construction of pBADaafDA plasmid

Inducible AafA expression was achieved by cloning *aafA* into the pBAD30 plasmid. To stabilize AafA expression, its chaperone *aafD* was also cloned bicistronically into the same plasmid. Briefly, a DNA fragment containing *aafD* and *aafA* was amplified by PCR from EAEC 042WT using Pfx platinum DNA polymerase (Invitrogen). Primers are shown in the Table S3. The PCR products were digested with SacI-HF and SalI-HF and ligated with Quick T4-DNA ligase (New England BioLabs) into pBAD30 plasmid previously digested with the same enzymes.

### Site-directed mutagenesis

Site-directed mutagenesis was performed following the QuikChange protocol (Stratagene, Cedar Creek, TX) and with the *Pfu*Turbo (Stratagene) high-fidelity polymerase. For each reaction, 25–50 ng of pBADaafDA or pET101D-AggAdsA-O104H4 plasmid was combined with 10 pmol of each of the complementary primers. Reactions were carried out according to the manufacturer's protocol. Primers used to generate the single point mutations are shown in Table S3. All constructs were verified by Sanger sequencing at the University of Virginia DNA Core Facility or Sequencing service of Turku Centre for Biotechnology. EAEC042*aafa* and DH5α were transformed with mutated pBADaafDA and pET101D-AggAdsA-O104H4, respectively, by heat shock at 42°C, rescued with SOC media, and selectively grown on LB-agar with kanamycin (pBADaafDA) or ampicillin (pET101D-AggAdsA-O104H4).

### SDS-PAGE and immunoblotting

Protein analysis of AafA constructs harboring site mutations was performed by immunoblotting. EAEC042*aafA* transformed with pBADaafDA harboring site mutations was grown until OD600 = 0.6, then induced with 2% arabinose until an OD600 = 1.2. 1×10^7^ cells were resuspended in Laemmlii buffer, boiled and proteins were separated by 4–15% gradient acrylamide SDS-PAGE and transferred by 1 h at 100 V in Towbin's buffer onto nitrocellulose membranes (BioRad, Hercules CA). Membranes were blocked with 5% skim milk in phosphate-buffered saline/Tween, incubated with 200 ng/mL primary anti-AafA rabbit polyclonal antibody followed by 40 ng/mL of secondary HRP-conjugated anti-rabbit antibody (KPL, Gaithersburg, MD). Results were visualized directly on nitrocellulose membranes after exposure with TMB membrane peroxidase substrate (KPL, Gaithersburg, MD. USA).

### Biofilm quantification

Biofilm formation was measured as previously described [12], [13]. Briefly, bacteria were grown at 37°C overnight in LB in 24-well plates and induced with 0.2% arabinose. After washing the substratum with PBS and fixation with 10% (vol/vol) formalin for 10 minutes, bacteria were stained with 0.5% crystal violet for 5 minutes and solubilized with 70% ethanol for 5 minutes. The resulting solution was transferred to a microtiter plate where absorbance was read at 450 nm.

### Bacterial binding to fibronectin

Quantification of bacterial binding to fibronectin was performed as previously described [23]. Briefly, wells of microtiter plates were coated with a solution of 25 µg/ml of Fn protein in 100 mM Tris-HCl buffer, pH 8.0, overnight at 4°C. Plates were washed 5 times with phosphate-buffered saline (PBS) to remove unbound protein and blocked with 5% milk in PBS for 4 h at 4°C. Wells were then washed five times prior to the addition of the bacteria. 1 ml Dulbecco's modified Eagle's medium (DMEM)/0.5% glucose medium containing 1×10^8^ bacteria at 37°C for 4 h were added to the wells. After the wells were washed 5 times with PBS, the bacterial cells that adhered to the wells were collected by scraping them into PBS with 0.1% (vol/vol) Triton X-100; serial dilutions were plated onto LB agar plates supplemented with ampicillin. The number of adherent bacteria was determined by counting the resulting colonies in duplicate.

### Immunofluorescence microscopy

Surface expression of AafA derivatives on EAEC042 was evaluated by indirect immunofluorescence assay (IFA). After induction of the bacterial strains with 0.2% arabinose, cells were harvested, washed twice with PBS, and incubated with a polyclonal anti-aafA antibody (2 µg/ml) in PBS plus 1% bovine serum albumin for 1h at room temperature with agitation. Cells were washed twice with PBS and incubated with a goat anti-rabbit IgG-fluorescein isothiocyanate (FITC) conjugate (10 µg/ml) for 30 min at room temperature in the dark. The washings were repeated, and the samples were resuspended in DAPI (4 mg/liter), and applied to slides for viewing by fluorescence microscopy.

### Negative stain electron microscopy

2 µl samples of sheared AAF/I fimbrae at ∼100 µg/ml and ∼10 µg/ml were applied to glow-discharged continuous-carbon-coated copper grids (Agar Scientific, UK), washed with 30 µl 2% (w/v) uranyl acetate, blotted and air dried. Microscopy was performed using a Philips CM200 FEG electron microscope.

### SPR binding assay

A Biacore X100 system (GE Healthcare) was used for all biosensor experiments. Fibronectin (Sigma) (approximately 1800 resonance units (RU)) was immobilized on flow cell 2 of a CM5 Sensor Chip by amine coupling using an Amine Coupling Kit (GE Healthcare). To record the association and dissociation curves, varying concentrations of subunit were injected into flow cell 2 of the chip for 3 min followed by flushing of the cell with 10 mM HEPES, pH 7.4, 150 mM NaCl, 3 mM EDTA, 0.005% Tween 20 (HBS-EP) for 3 min at a flow rate of 10 ml min^−1^. Identical samples were injected over a control flow cell to determine non-specific binding, which was subtracted from the experimental curves. The sensor chip was regenerated with 0.1% SDS. The equilibrium constants were determined by applying a one receptor binding model, using the Biacore X100 evaluation software and Simfit/HLFIT program.

### Statistical analysis

Statistical significance between means was analyzed using the unpaired Student's *t* test with a threshold *P* value of 0.05. Values are expressed as the means of three experiments with one standard deviation errors.

## Supporting Information

Figure S1 The primary sequence alignment of major subunits from AAF and Afa/Dr families. The invariant and conserved positions are shaded in cyan and yellow, respectively.
(TIFF)

Figure S2 Protein sequences of designed constructs. Blue, signal peptide; orange, His-tag; green, linker sequence; red, donor strand sequence.
(TIFF)

Figure S3 Dynamic structure of AAF subunits. (A) NMR Relaxation properties of AafAdsA. Top panel: Black: Random Coil Index (RCI) predicted by TALOS+ based on AafA-dsc backbone chemical shifts. Region from residue number 125 to 135 shows high flexibility. Red: RMSD of each AafA residue generated by ARIA/CNS. Middle panel: T_2_ relaxation analysis of AafA-dsc. Bottom panel: ^1^H-^15^N Heteronuclear NOE spectrum of AafAdsA. (B) Superimposition of the 10 best NMR structures of AafAdsA. (C) Structural superposition of the eight independently refined molecules from the asymmetric unit of the AggBdscA crystal. In A and C, the donor strand and linker sequence are shown in yellow and blue, respectively. The flexible N-terminal residues in the NMR structure are colored magenta.
(TIFF)

Figure S4 Structural superposition of AggAdsA (magenta), AafAdsA (marine), and AfaEdsE (pdb: 2ixq) [35] (green) (stereo view) showing structurally variable segments.
(TIFF)

Figure S5 Localization of key residues conserved across the major subunits of both AAF/I-III and AFA/Dr families (shaded in Fig. S1). These include the invariant Cys17, Cys49, Gly127 and Tyr129; the highly conserved Leu11 in the donor strand and Asp97, which is Asn in AAF/I from EAEC strain 17. Also shown are residues conserved within AAF/I-III major subunits only (i.e. not present in the AFA/Dr family), which include Lys55, Trp59, Gly60, Thr80 and Leu81 residues (from those shaded blue in Fig. 1). Side-chains are shown as balls on sticks on the structure of AggAdsA as a stereo view. Residues are numbered according to the sequence of AggA.
(TIFF)

Figure S6 Modelling of AAF fibers (A) Molecular surface rendering of a model for AAF fibers. Fragments containing tip minor subunits and four major subunits are shown. Conserved surface residues (Fig. 4) are painted in red. (B) Structural superposition of the Caf1':Caf1" fragment of the crystal structure of the Caf1M:Caf1':Caf1" mini-fiber of the F1 antigen (PDB accession number 1Z9S) and solution structure of the AfaDdsE-AfaEdsE fusion protein representing the tip complex of Afa-III fimbriae (dsE, donor strand of AfaE, PDB accession number 2IXQ). The Caf1':Caf1" fragment is shown in magenta except the donor strand, which is shown in red. The AfaDdsE-AfaEdsE fusion is painted in green, except the dsE donor strand complementing the AfaD subunits (blue) and the linker sequence connecting dsE to AfaD (cyan). The N and C termini of protein chains are labeled. Note that the angle between adjacent subunits in the F1 and Afa-III fibers differs by ∼25°.
(TIFF)

Figure S7 Fragment of the tip complex in AAF/I demonstrating the difference in the topology of disulfide bonds (balls on sticks) in the minor (AggB, green) and major (AggA, cyan) subunits (cartoon diagram, stereo view). Note that in AggA, the disulfide bond connects the α helix in the BC_1_ loop with the donor strand linker, whereas in AggB, the disulfide bond connects the BC_1_ loop with the end of strand F.
(TIFF)

Figure S8 Biacore analysis of AggAdsA (A) and AggBdsA (B) binding to fibronectin. Left panels: SPR sensograms recorded for different concentration of AggAdsA or AggBdsA. Right panels: Saturation curves (one binding site model).
(TIFF)

Figure S9 Expression of AafA harboring site mutations in EAEC. Protein analysis of AafA constructs harboring site mutations was performed by immunobloting. EAEC *aafA* mutant, was transformed with pBADaafDA harboring site mutations and grown until OD600 = 0.6, then induced with 2% arabinose until an OD600 = 1.2 was reached. 1×10^7^ cells were resuspended in Laemmli buffer, boiled and proteins were separated by 4–15% gradient SDS-PAGE and transferred onto nitrocellulose membranes. Membranes were probed with an anti-AafA rabbit polyclonal antibody followed by HRP-conjugated anti-rabbit antibody. All residues were mutated to alanine except where noted.
(TIFF)

Figure S10 Comparison of structure and stability of the wild type (WT) AggAdsA and AggAdsA carrying three mutations: Lys73Ala, Lys76Ala, and Lys78Ala. (A) Far UV circular dichroism (CD) spectra of the wild type AggAdsA at 20 (solid line) and 80°C (dashed line) and mutant AggAdsA at 20°C (dotted line). 20-40 g/l samples of the proteins in 10 mM Hepes, pH 7.4 and 50 mM NaCl were diluted to the concentration of 3 g/l with 20 mM phosphate buffer, pH 7.0, and CD spectra were recorded on a J-810 dichrograph (Jasco) equipped with a PTC-423S temperature control system in a 0.01 cm quartz cuvette. (B) Temperature stability of the wild type and mutant AggAdsA. Temperature dependence of the CD signal was recorded at a heating rate of 1°C min^−1^ at 218 nm for the wild type AggAdsA and at 230 nm for the mutant in a 0.2 cm cuvette. The protein concentration was 0.21 g/ml. The data were normalized and fitted to a two-state denaturation process. The transition temperatures for the wild type and mutant AggAdsA have similar values, 66.8±0.4 and 65.4±0.2°C, respectively, suggesting that the introduced mutations did not affect protein stability.
(TIFF)

Figure S11 Bovine serum albumin (BSA) does not affect the AggAdsA binding to fibronectin. SPR sensograms for the binding of AggAdsA and fibronectin were recorded at 40 µM concentration of AggAdsA in the absence (black solid line) and presence of 240 µM BSA (red dashed line).
(TIFF)

Table S1 NMR structural constraints and structure statistics for AafAdsA.
(PDF)

Table S2 Diffraction data and refinement statistics.
(PDF)

Table S3 Primers used in the study.
(PDF)
