# Supplementary figures and images for: Structural Insight into Host Recognition by Aggregative Adherence Fimbriae of Enteroaggregative Escherichia coli
Source: PLoS Pathog. 2014 Sep 18;10(9):e1004404. doi: 10.1371/journal.ppat.1004404 (PMC4169507; doi:10.1371/journal.ppat.1004404)

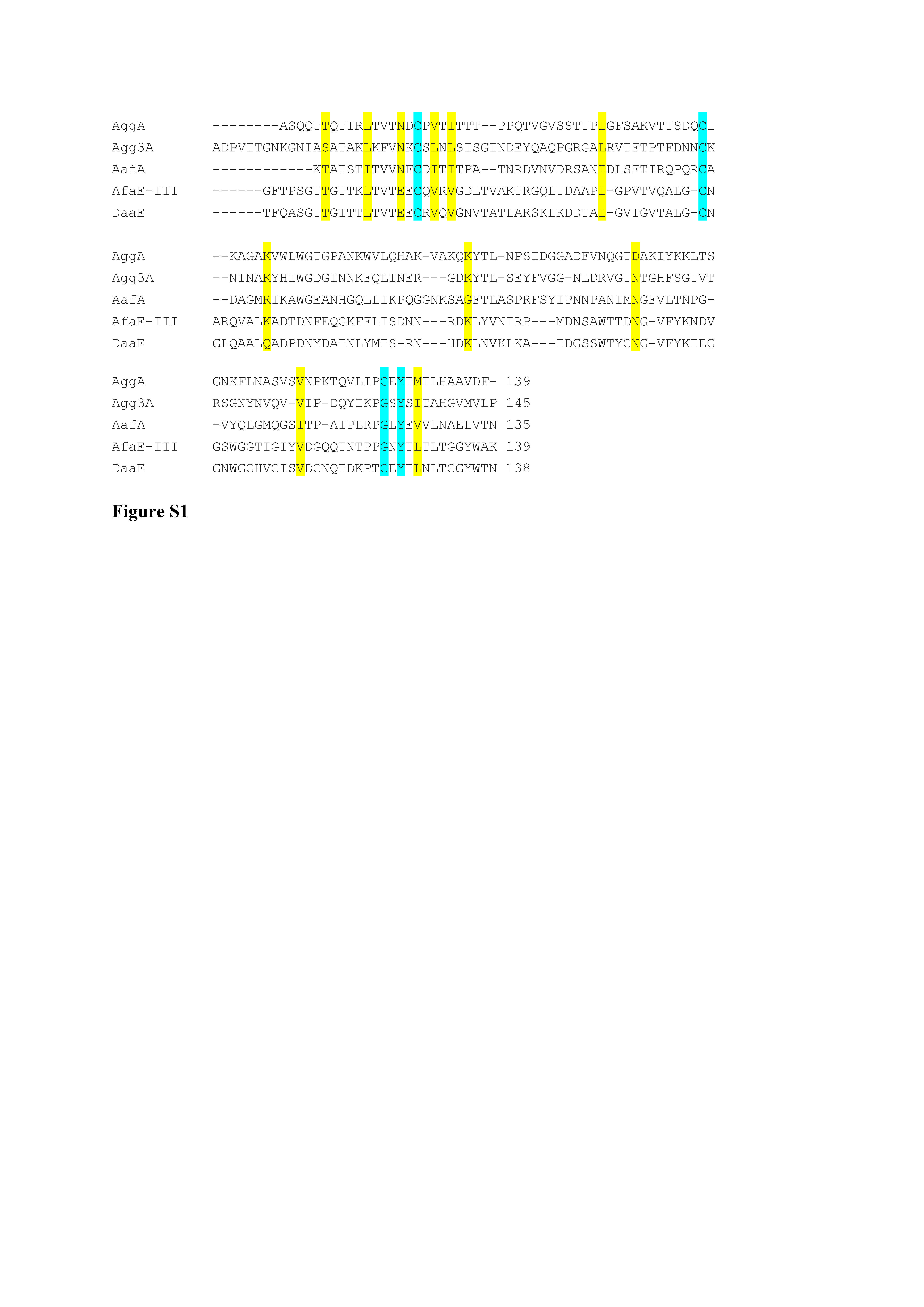

Supplement: Figure S1 — The primary sequence alignment of major subunits from AAF and Afa/Dr families. The invariant and conserved positions are shaded in cyan and yellow, respectively. (TIFF) [file ppat.1004404.s001.tiff]

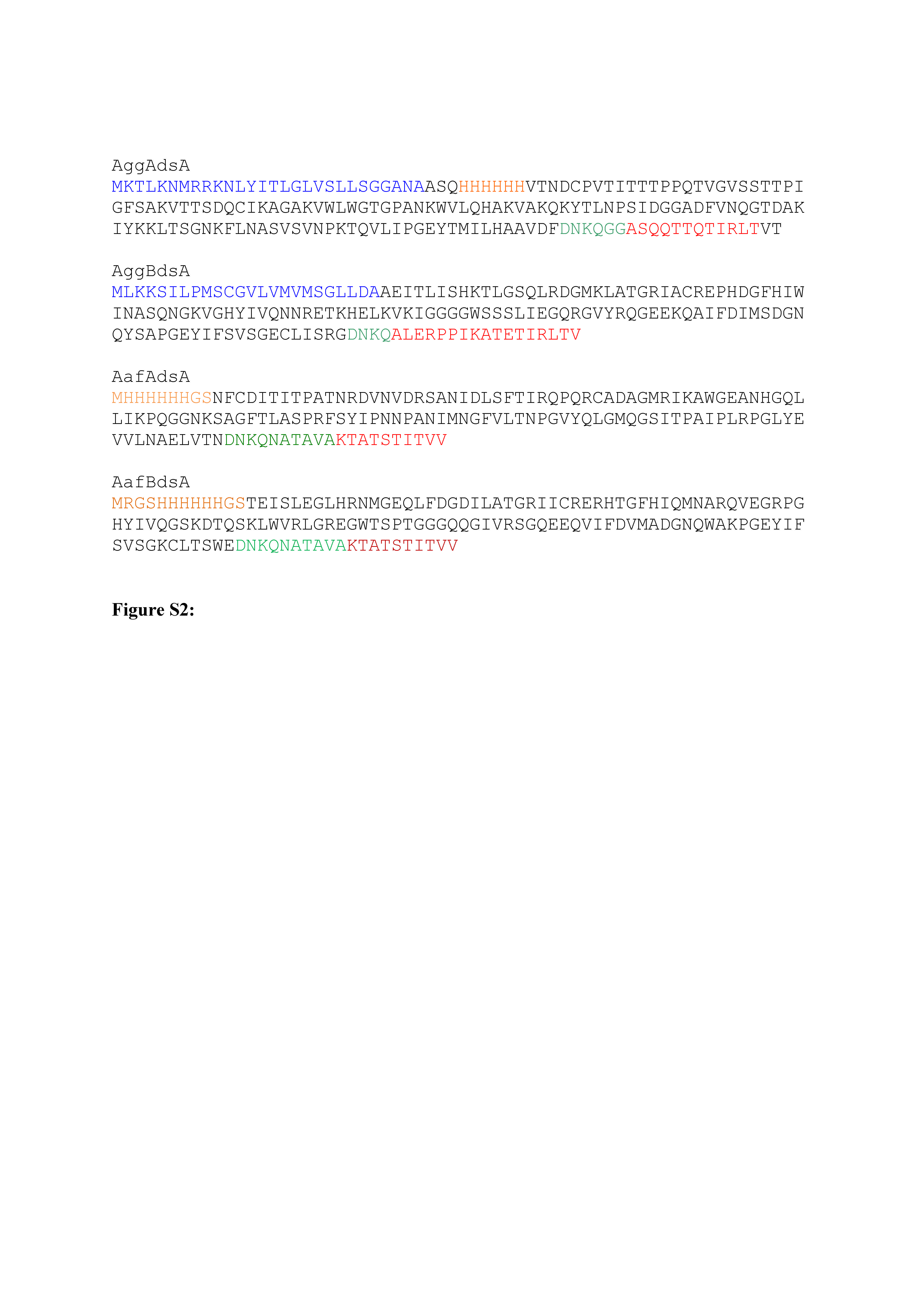

Supplement: Figure S2 — Protein sequences of designed constructs. Blue, signal peptide; orange, His-tag; green, linker sequence; red, donor strand sequence. (TIFF) [file ppat.1004404.s002.tiff]

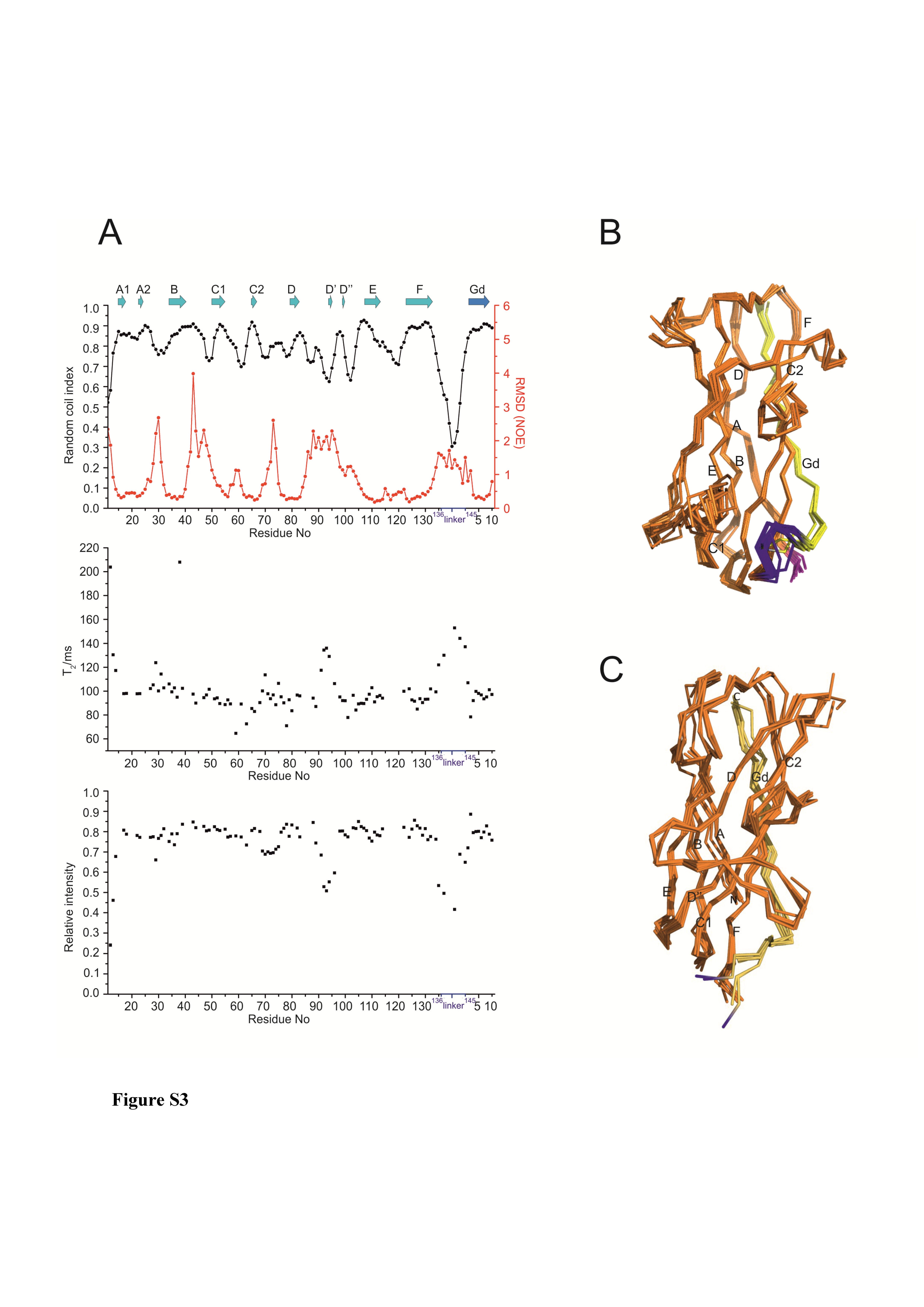

Supplement: Figure S3 — Dynamic structure of AAF subunits. (A) NMR Relaxation properties of AafAdsA. Top panel: Black: Random Coil Index (RCI) predicted by TALOS+ based on AafA-dsc backbone chemical shifts. Region from residue number 125 to 135 shows high flexibility. Red: RMSD of each AafA residue generated by ARIA/CNS. Middle panel: T2 relaxation analysis of AafA-dsc. Bottom panel: 1H-15N Heteronuclear NOE spectrum of AafAdsA. (B) Superimposition of the 10 best NMR structures of AafAdsA. (C) Structural superposition of the eight independently refined molecules from the asymmetric unit of the AggBdscA crystal. In A and C, the donor strand and linker sequence are shown in yellow and blue, respectively. The flexible N-terminal residues in the NMR structure are colored magenta. (TIFF) [file ppat.1004404.s003.tiff]

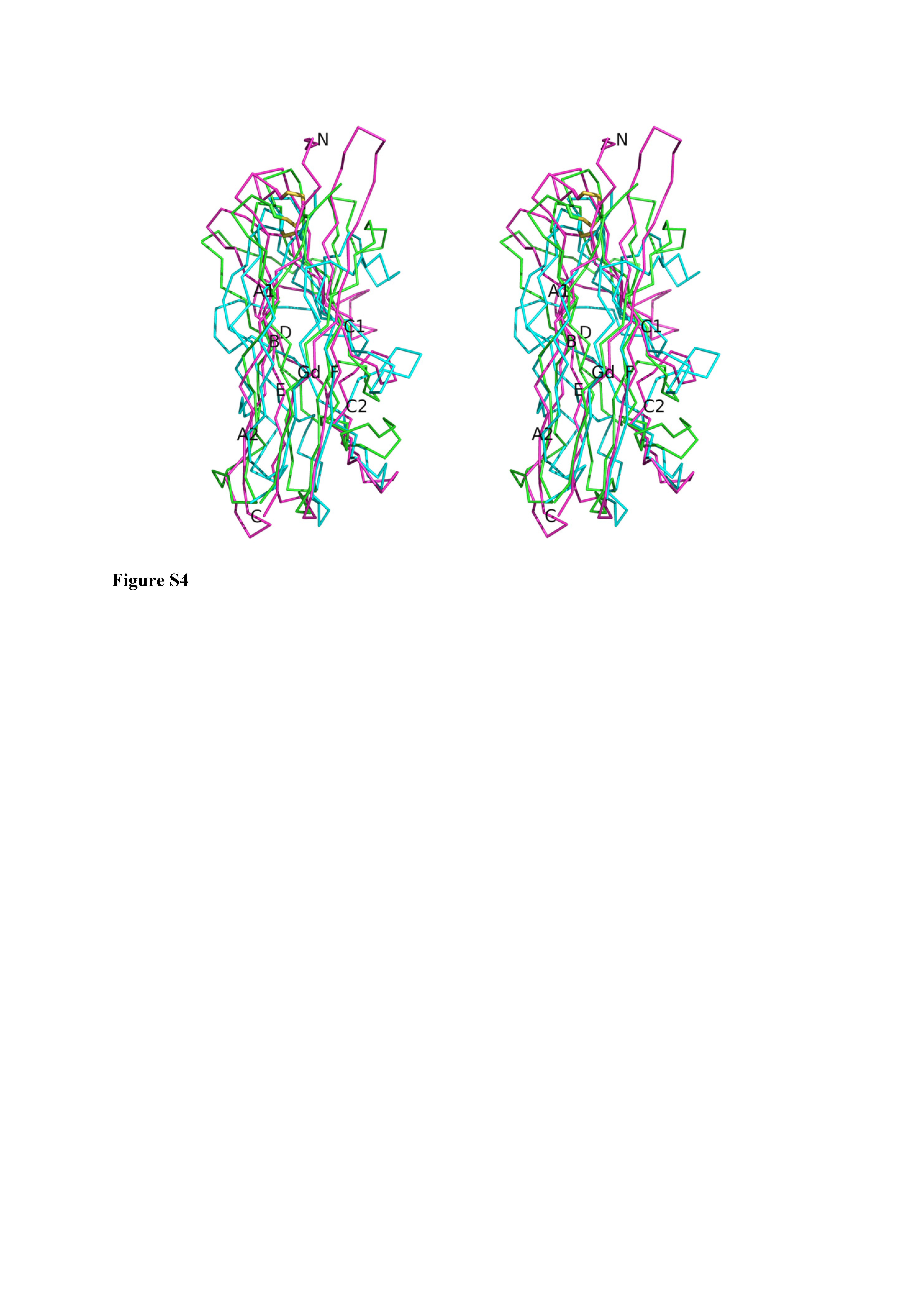

Supplement: Figure S4 — Structural superposition of AggAdsA (magenta), AafAdsA (marine), and AfaEdsE (pdb: 2ixq) [35] (green) (stereo view) showing structurally variable segments. (TIFF) [file ppat.1004404.s004.tiff]

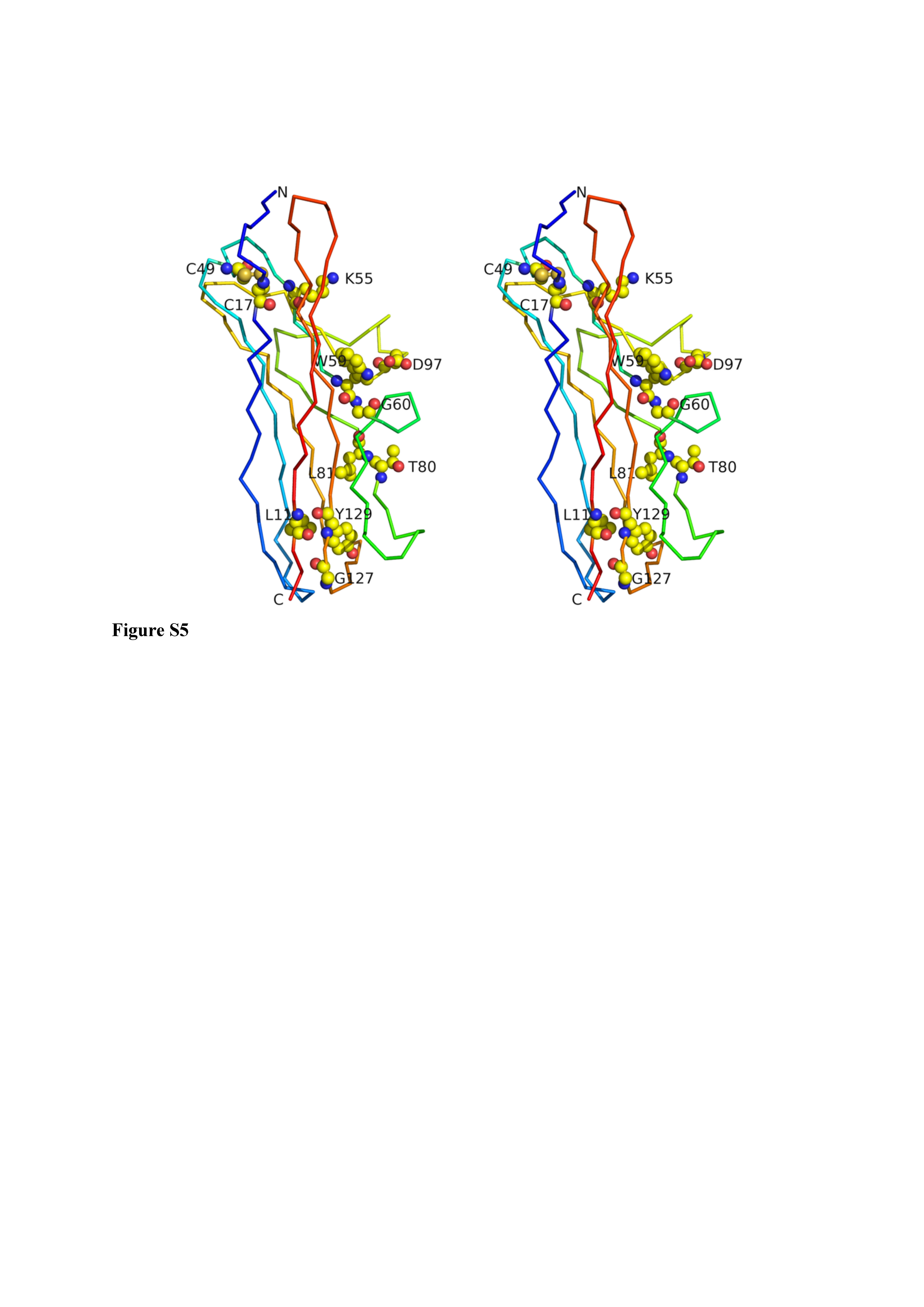

Supplement: Figure S5 — Localization of key residues conserved across the major subunits of both AAF/I-III and AFA/Dr families (shaded in Fig. S1). These include the invariant Cys17, Cys49, Gly127 and Tyr129; the highly conserved Leu11 in the donor strand and Asp97, which is Asn in AAF/I from EAEC strain 17. Also shown are residues conserved within AAF/I-III major subunits only (i.e. not present in the AFA/Dr family), which include Lys55, Trp59, Gly60, Thr80 and Leu81 residues (from those shaded blue in Fig. 1). Side-chains are shown as balls on sticks on the structure of AggAdsA as a stereo view. Residues are numbered according to the sequence of AggA. (TIFF) [file ppat.1004404.s005.tiff]

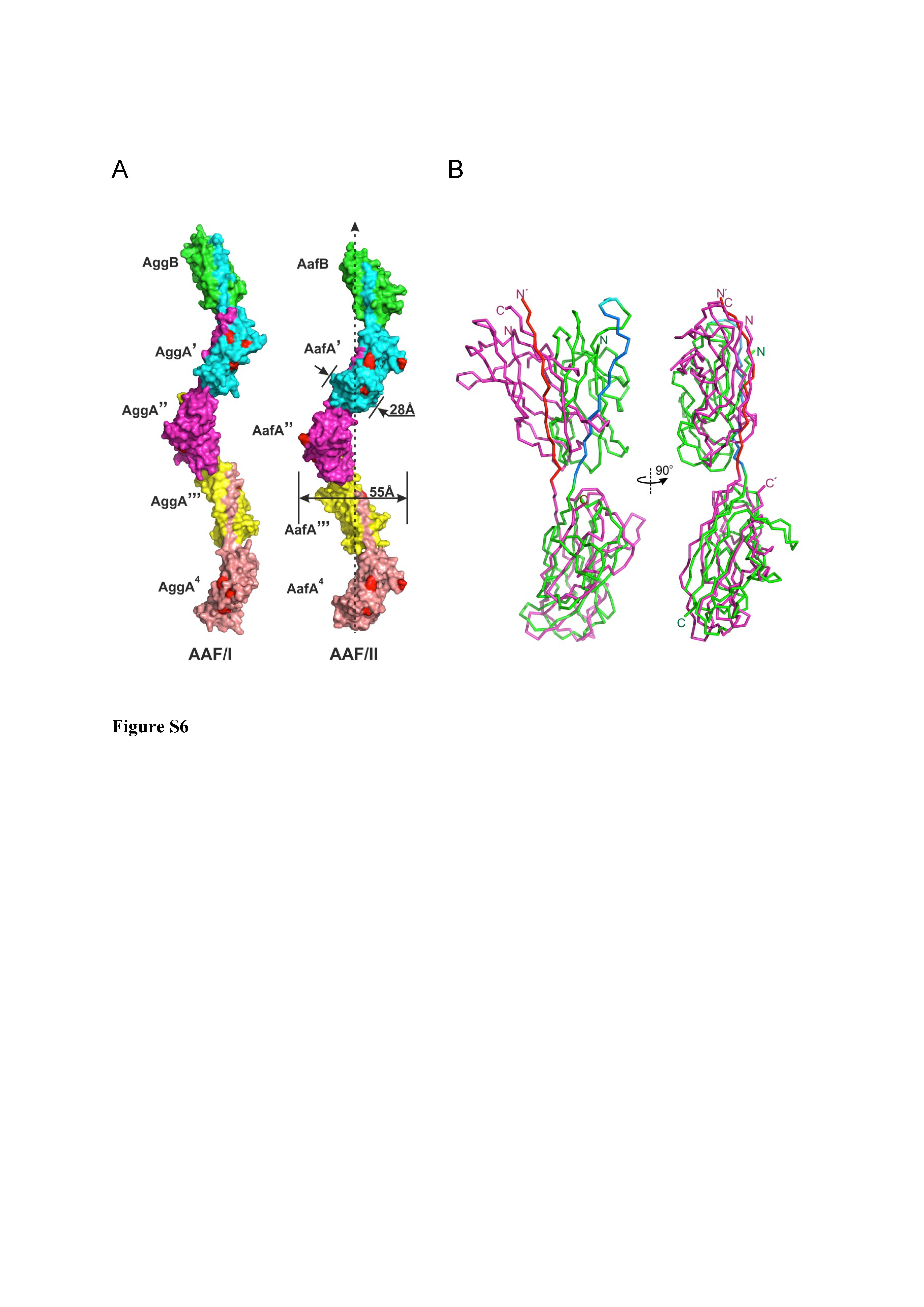

Supplement: Figure S6 — Modelling of AAF fibers (A) Molecular surface rendering of a model for AAF fibers. Fragments containing tip minor subunits and four major subunits are shown. Conserved surface residues (Fig. 4) are painted in red. (B) Structural superposition of the Caf1':Caf1" fragment of the crystal structure of the Caf1M:Caf1':Caf1" mini-fiber of the F1 antigen (PDB accession number 1Z9S) and solution structure of the AfaDdsE-AfaEdsE fusion protein representing the tip complex of Afa-III fimbriae (dsE, donor strand of AfaE, PDB accession number 2IXQ). The Caf1':Caf1" fragment is shown in magenta except the donor strand, which is shown in red. The AfaDdsE-AfaEdsE fusion is painted in green, except the dsE donor strand complementing the AfaD subunits (blue) and the linker sequence connecting dsE to AfaD (cyan). The N and C termini of protein chains are labeled. Note that the angle between adjacent subunits in the F1 and Afa-III fibers differs by ∼25°. (TIFF) [file ppat.1004404.s006.tiff]

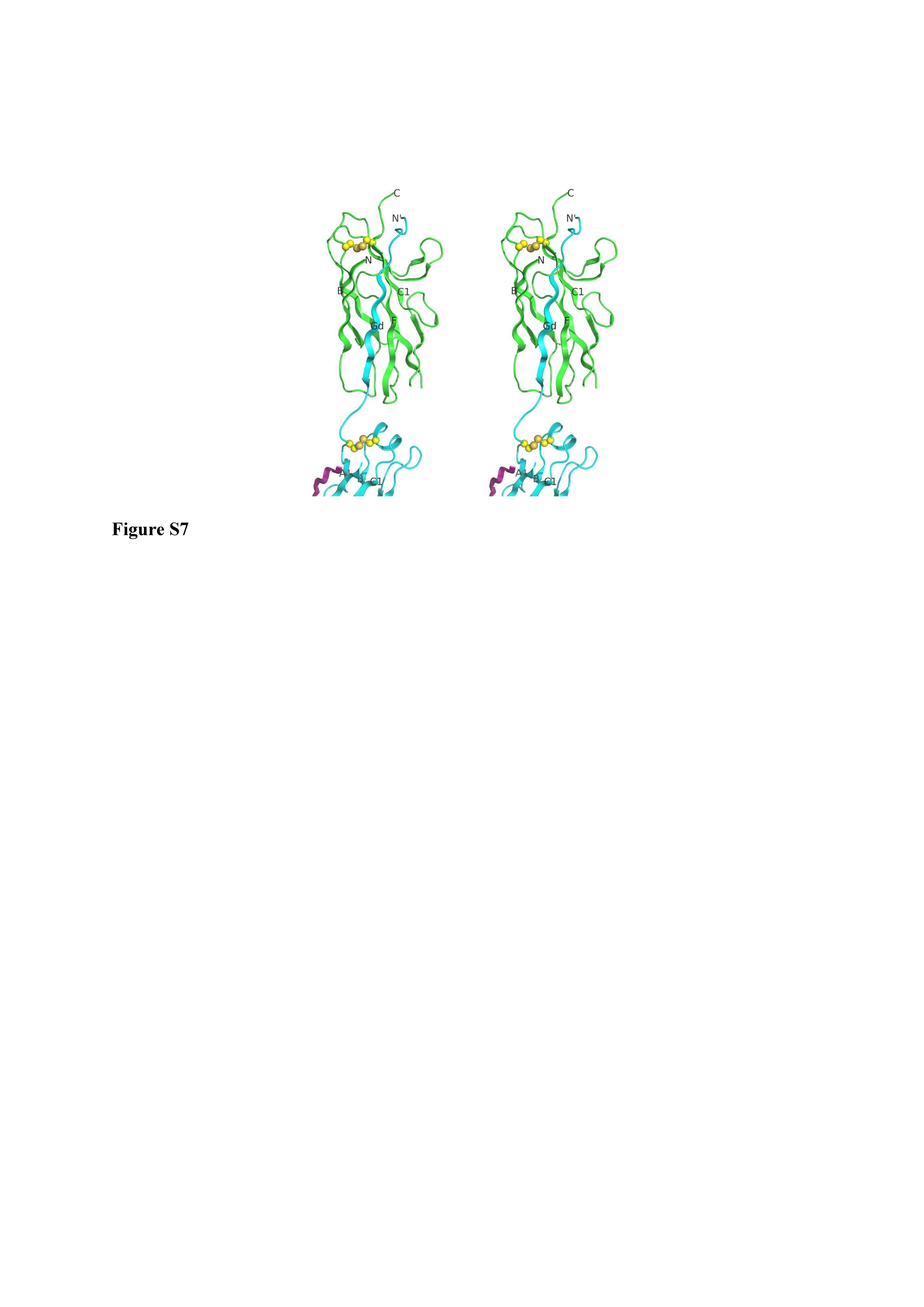

Supplement: Figure S7 — Fragment of the tip complex in AAF/I demonstrating the difference in the topology of disulfide bonds (balls on sticks) in the minor (AggB, green) and major (AggA, cyan) subunits (cartoon diagram, stereo view). Note that in AggA, the disulfide bond connects the α helix in the BC1 loop with the donor strand linker, whereas in AggB, the disulfide bond connects the BC1 loop with the end of strand F. (TIFF) [file ppat.1004404.s007.tiff]

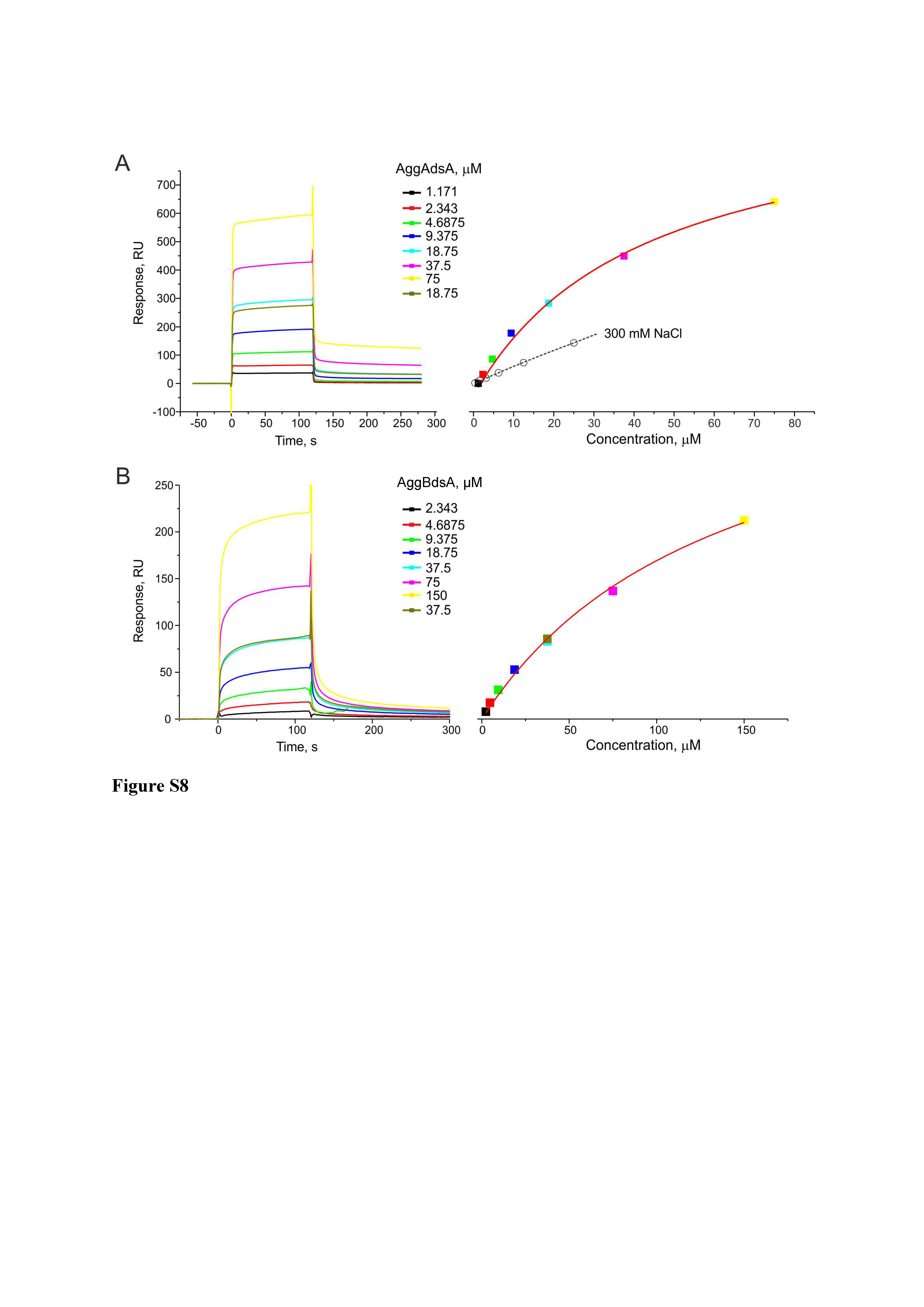

Supplement: Figure S8 — Biacore analysis of AggAdsA (A) and AggBdsA (B) binding to fibronectin. Left panels: SPR sensograms recorded for different concentration of AggAdsA or AggBdsA. Right panels: Saturation curves (one binding site model). (TIFF) [file ppat.1004404.s008.tiff]

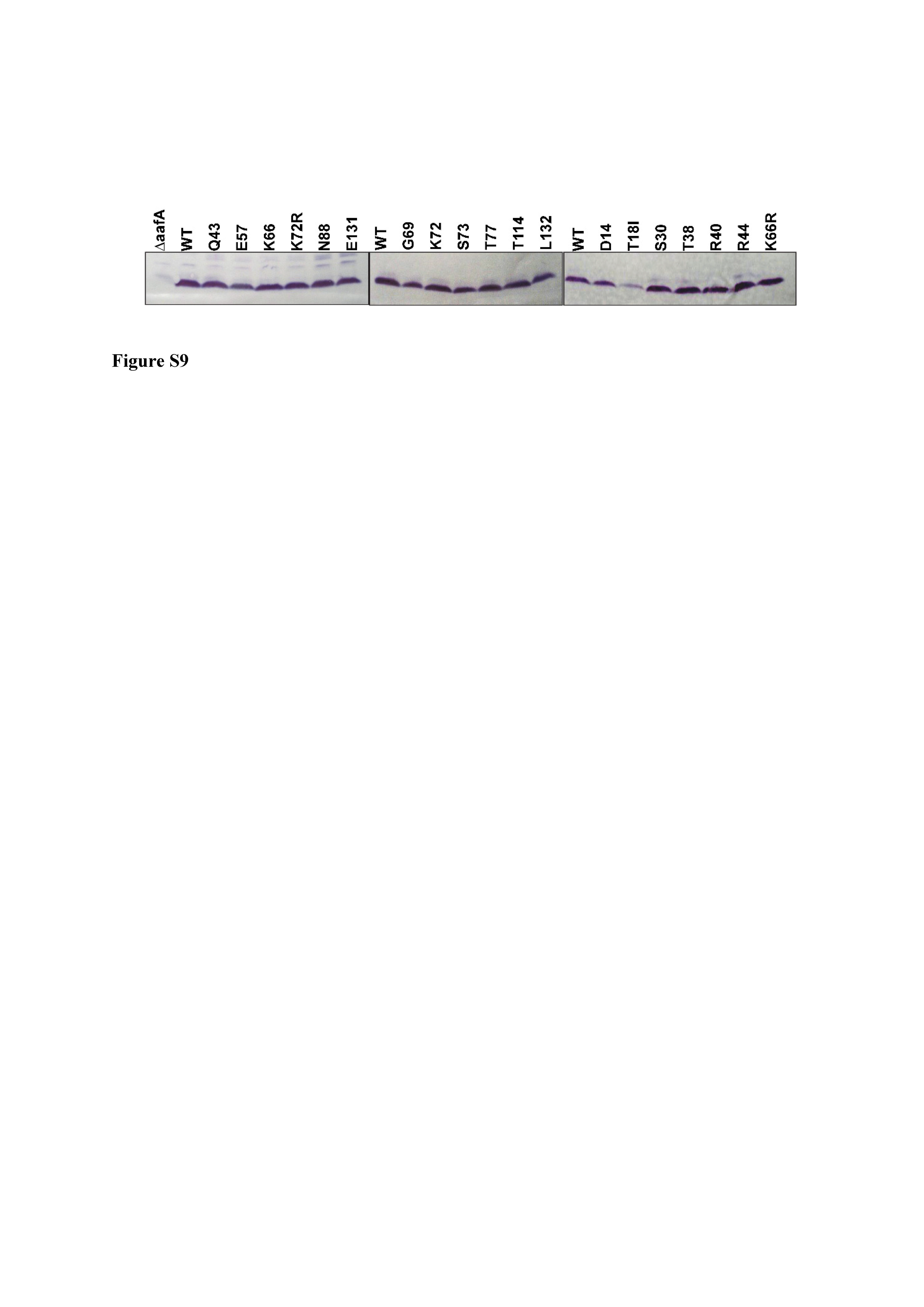

Supplement: Figure S9 — Expression of AafA harboring site mutations in EAEC. Protein analysis of AafA constructs harboring site mutations was performed by immunobloting. EAEC aafA mutant, was transformed with pBADaafDA harboring site mutations and grown until OD600 = 0.6, then induced with 2% arabinose until an OD600 = 1.2 was reached. 1×10^7^ cells were resuspended in Laemmli buffer, boiled and proteins were separated by 4–15% gradient SDS-PAGE and transferred onto nitrocellulose membranes. Membranes were probed with an anti-AafA rabbit polyclonal antibody followed by HRP-conjugated anti-rabbit antibody. All residues were mutated to alanine except where noted. (TIFF) [file ppat.1004404.s009.tiff]

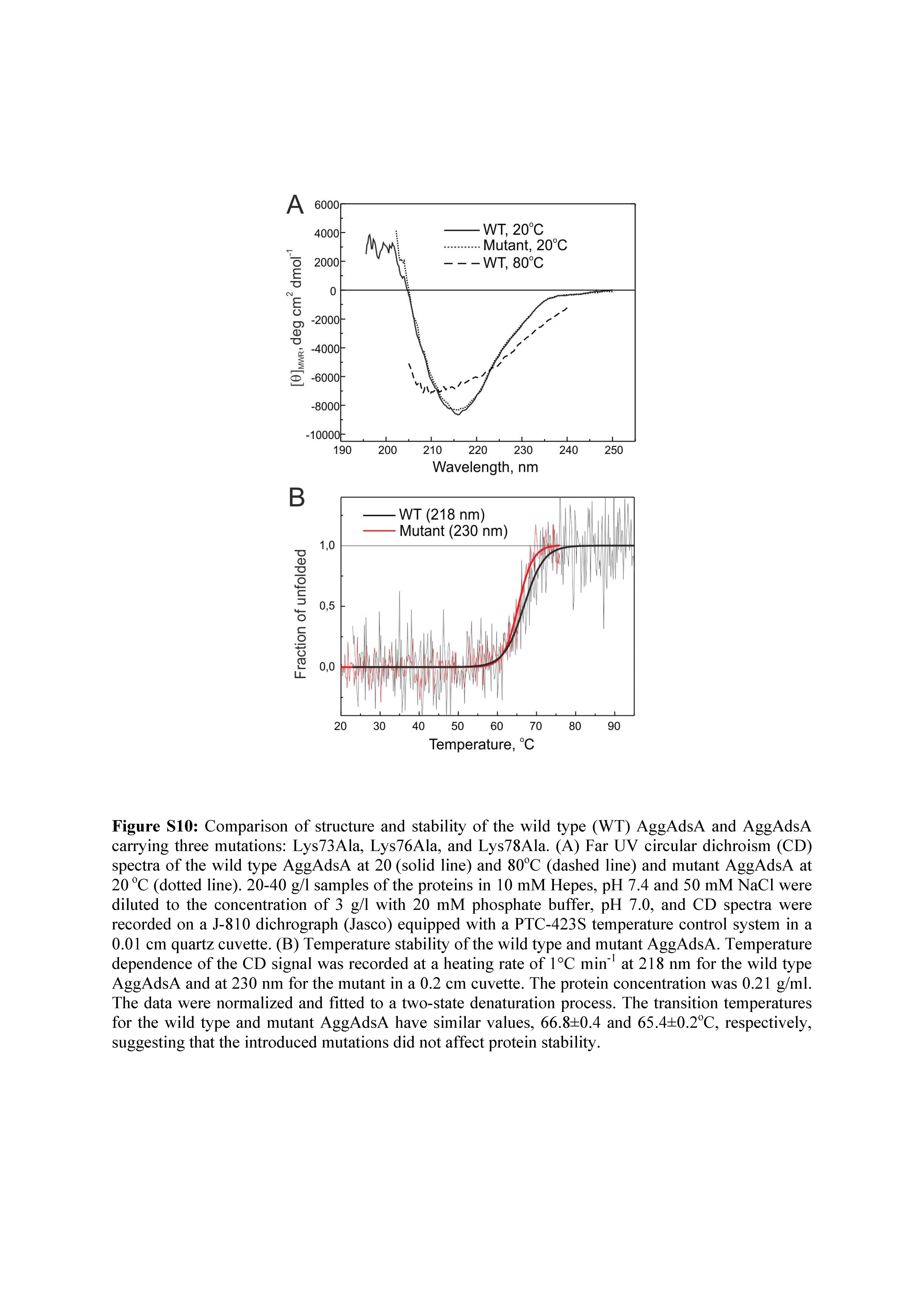

Supplement: Figure S10 — Comparison of structure and stability of the wild type (WT) AggAdsA and AggAdsA carrying three mutations: Lys73Ala, Lys76Ala, and Lys78Ala. (A) Far UV circular dichroism (CD) spectra of the wild type AggAdsA at 20 (solid line) and 80°C (dashed line) and mutant AggAdsA at 20°C (dotted line). 20-40 g/l samples of the proteins in 10 mM Hepes, pH 7.4 and 50 mM NaCl were diluted to the concentration of 3 g/l with 20 mM phosphate buffer, pH 7.0, and CD spectra were recorded on a J-810 dichrograph (Jasco) equipped with a PTC-423S temperature control system in a 0.01 cm quartz cuvette. (B) Temperature stability of the wild type and mutant AggAdsA. Temperature dependence of the CD signal was recorded at a heating rate of 1°C min−1 at 218 nm for the wild type AggAdsA and at 230 nm for the mutant in a 0.2 cm cuvette. The protein concentration was 0.21 g/ml. The data were normalized and fitted to a two-state denaturation process. The transition temperatures for the wild type and mutant AggAdsA have similar values, 66.8±0.4 and 65.4±0.2°C, respectively, suggesting that the introduced mutations did not affect protein stability. (TIFF) [file ppat.1004404.s010.tiff]

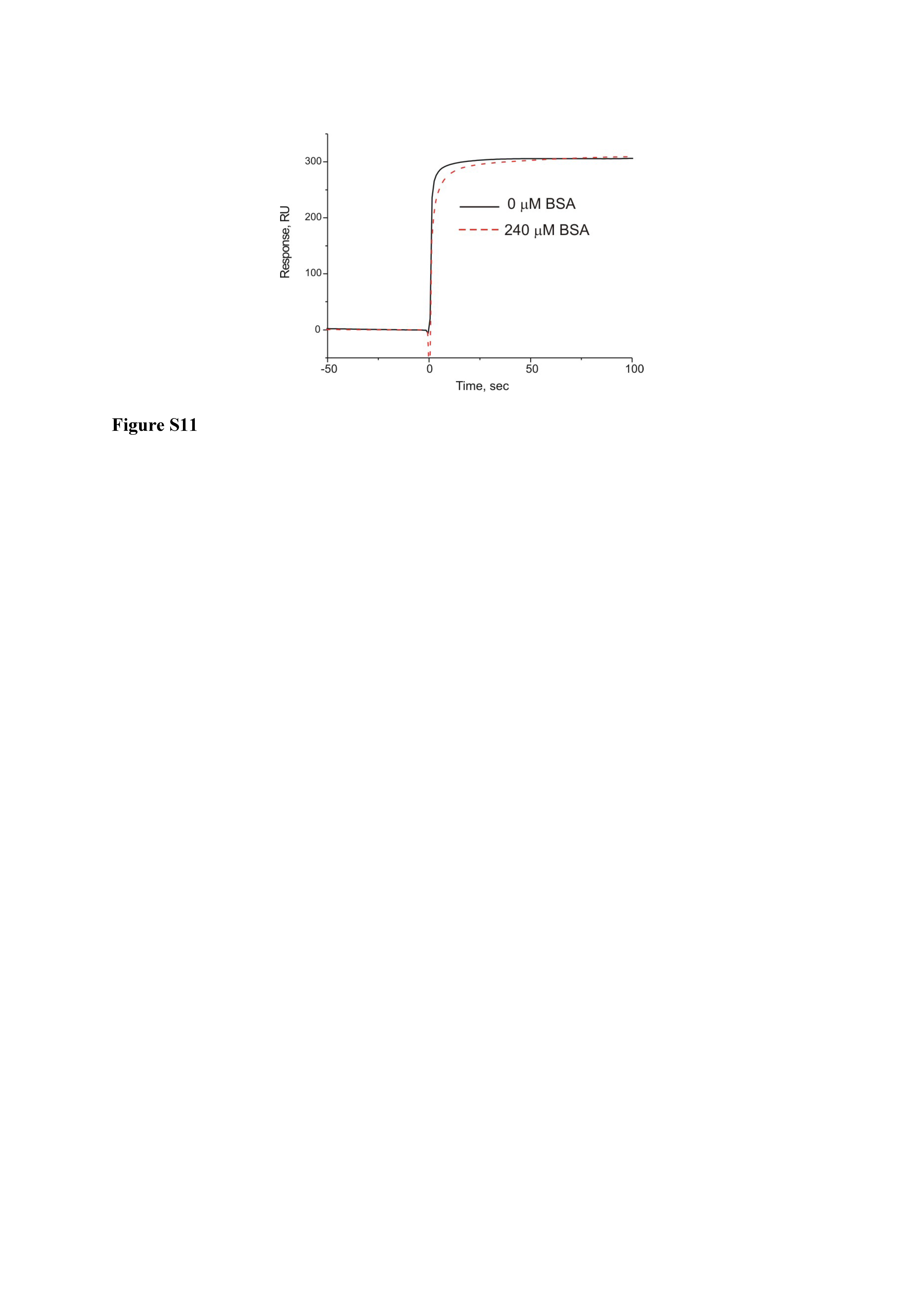

Supplement: Figure S11 — Bovine serum albumin (BSA) does not affect the AggAdsA binding to fibronectin. SPR sensograms for the binding of AggAdsA and fibronectin were recorded at 40 µM concentration of AggAdsA in the absence (black solid line) and presence of 240 µM BSA (red dashed line). (TIFF) [file ppat.1004404.s011.tiff]
