# Supplementary material for: Structural Insight into Host Recognition by Aggregative Adherence Fimbriae of Enteroaggregative Escherichia coli
Source: PLoS Pathog. 2014 Sep 18;10(9):e1004404. doi: 10.1371/journal.ppat.1004404 (PMC4169507; doi:10.1371/journal.ppat.1004404)
Supplement: Table S1 — NMR structural constraints and structure statistics for AafAdsA. (PDF) [file ppat.1004404.s012.pdf]

Table S1

**Summary of structural constraints and structure statistics**	
**Experimental restraints**	
Distance restraints	2721
Intra-residue (|i-j| = 0)	686
Sequential (|i-j| = 1)	415
Medium-range (2 ≤ |i-j| ≤ 5)	135
Long-range (|i-j| > 5)	682
Ambiguous	803
Total	
Dihedral angle restraints (φ/ψ)	124
**Restraints statistics**†	
RMS of distance violations (Å)	0.1363 ± 0.0293
RMS of dihedral violations (°)	4.6516 ± 0.7490
**Structural quality**†	
Ramachandran statistics¶	
Most favoured regions (%)	89
Allowed regions (%)	8
Disallowed regions (%)	3
**Coordinates precision***	
All backbone atoms (Å)	0.32 ± 0.1
All heavy atoms (Å)	0.67 ± 0.008

† Average values and standard deviations over the 10 lowest-energy conformers

¶ Percentage of residues in the Ramachandran plot regions determined by MOLPROBITY [51]

* Average root mean square deviation (RMSD) over the 10 lowest-energy conformers' atomic coordinates with respect to the average structure.
