# Supplementary material for: Structural Insight into Host Recognition by Aggregative Adherence Fimbriae of Enteroaggregative Escherichia coli
Source: PLoS Pathog. 2014 Sep 18;10(9):e1004404. doi: 10.1371/journal.ppat.1004404 (PMC4169507; doi:10.1371/journal.ppat.1004404)
Supplement: Table S2 — Diffraction data and refinement statistics. (PDF) [file ppat.1004404.s013.pdf]

Table S2

Crystal parameters	AafB	AggA	AggB
Space group	$P6_122$	$C222_1$	$P2_12_12$
Cell dimensions	*a*=*b*=81.76, *c*=222.70	a=77,832, b=80.172, c=91.42	a=130.80, b=163.97, c=58.63
Number of molecules per assymetric unit	2	2	8
**Data collection**			
Beamline	DLS I24	BM-14	ID23-1
Wavelength (Å)	0.97780	0.954	0.98034
Resolution (Å)	43.77-3.00 (3.16-3.00)	47.66-1.55 (1.63-1.55)	58.63-2.40 (2.53-2.40)
Unique observations	9081 (768)	40475 (5758)	50214 (7238)
R_{merge}	0.111 (0.374)	0.038 (0.787)	0.062 (0.565)
$<I>/\sigma I$	6.5 (2.6)	19.0 (2.1)	14.8 (2.8)
Completeness (%)	97.0 (98.2)	97.2 (95.8)	99.9 (99.9)
Redundancy	2.9 (2.9)	4.2 (4.3)	5.2 (5.4)
Overall *B* factor from Wilson plot (Å)	74.4	22.9	55.1
**Refinement**			
$R_{\mathrm{work}}/R_{\mathrm{free}}$ (%)	21.8/24.6	15.6/23.2	22.8/27.2
Number of protein residues	263	155	142
Number of ligands/ions	1 sulphate ion, 1 acetate ion	2 glycerol molecules	0
rmsd stereochemistry			
Bond lengths (Å)	0.009	0.008	0.009
Bond angles (°)	1.181	1.344	1.352
Ramachandran analysis*			
Residues in outlier regions	0	0	0.3%
Residues in favoured regions	95.5%	98%	93.0%
Residues in allowed regions	100%	100%	99.7%

Values for high resolution shells in parenthesis.
$R_{merge} = \Sigma_h \Sigma_i |I(h)_i - (I)| / \Sigma_h \Sigma_i I(h)_i$, where $I(h)$ is the intensity of a reflection h, Σ_h is the sum over all reflections and Σ_i is the sum over i measurements of reflection h.
$R_{work} = \Sigma|F_O - F_C|/\Sigma F_O$ where F_O and F_C are the observed and calculated structure factors respectively. R_{free} is calculated for a test set of reflections randomly excluded from refinement.
R.m.s.d. stereochemistry is the deviation from ideal values. R.m.s.d. B-factors is deviation between bonded atoms.
*Output from Molprobity
