# Supplementary material for: Structural Insight into Host Recognition by Aggregative Adherence Fimbriae of Enteroaggregative Escherichia coli
Source: PLoS Pathog. 2014 Sep 18;10(9):e1004404. doi: 10.1371/journal.ppat.1004404 (PMC4169507; doi:10.1371/journal.ppat.1004404)
Supplement: Table S3 — Primers used in the study. (PDF) [file ppat.1004404.s014.pdf]

Table S3: Primers used in the study

Primer name	Sequence (5'-3')
pBADaafDA plasmid construction	
aafDA F	ccggaccggtaaggcgagctccccgtttttgggctaacaggaggaattaaccatgtataaaatg aaaatacggagaattc
aafA R	ttaaggtaaggtctcgtcgacaaaattaaagggcggcgagccgc
aafA mutagenesis	
D14A F	tcactgtagtgaataattgtgctataacgataacaccggctac
D14A R	gtagccggtgttatcgttatagcacaattattcactacagtga
T18I F	actgtagtgaataattgtgatataacgataataccggctacaaatcgt
T18I R	acgatttgtagccggtattatcgttatatcacaattattcactacagt
S30A F	gatgtcaacgttgacagggccgcaaatatcgacctgag
S30A R	ctcaggtcgatatttgcggccctgtcaacgttgacatc
R40A F	gcaaatatcgacctgagttttactattgcacaaccgcaacgct
R40A R	agcgttgcggttgtgcaatagtaaaactcaggtcgatatttgc
Q43A F	tttactattagacaaccggcacgctgcgctgatgctgg
Q43A F	ccagcatcagcgcagcgtgccggttgtctaatagtaaa
R44A F	gttttactattagacaaccgcaagcctgcgctgatgctgg
R44A R	ccagcatcagcgcaggcttgcggttgtctaatagtaaaac
E57A F	aataaaagcttgggggcaggcaatcacggtcaat
E57A R	attgaccgtgattgcctgcccccaagcttttatt
K66A F	gaaggcaatcacggtcaattactgatagcacctcaaggaggaa
K66A R	ttcctccttgaggtgctatcagtaattgaccgtgattgccttc
K66R F	gcaatcacggtcaattactgataagacctcaaggagga
K66R R	tcctccttgaggtcttatcagtaattgaccgtgattgc
G69A F	caattactgataaaacctcaagcaggaaataaatcagcaggattc
G69A R	gaatcctgctgatttatttcctgcttgaggttttatcagtaattg
K72A F	caattactgataaaacctcaaggaggaaatgcatcagcaggattcactctg
K72A R	cagagtgaatcctgctgatgcatttcctccttgaggttttatcagtaattg
K72R F	ttactgataaaacctcaaggaggaaatagatcagcaggattcac
K72R R	gtgaatcctgctgatctatttcctccttgaggttttatcagtaa
S73A F	actgataaaacctcaaggaggaaataaagcagcaggattcactc
S73A R	gagtgaatcctgctgctttatttcctccttgaggttttatcagt
T77A F	ataaatcagcaggattcgctctggcctctcctagg
T77A R	cctaggagaggccagagcgaatcctgctgatttat
N88A F	ctctcctaggttttcttacattccggctaatccaacaaacattatgaatgga
N88A R	tccattcataatgtttgttggattagccggaatgtaagaaaacctaggagag
T114A F	attaggaatgcagggctcaattgcaccggctatgc
T114A R	gcatagccggtgcaattgagccctgcattcctaat
E131A F	tatatgaagtagtattaaatgctgcgcttgtgacaaattaaatgctgag
E131A R	ctcagcatttaatttgtcacaagcgcagcatttaatactacttcatata
L132A F	atatgaagtagtattaaatgctgaggctgtgacaaattaaatgctgagcttg
L132A R	caagctcagcatttaatttgtcacagcctcagcatttaatactacttcatat

**Supplementary Table 3:** continued	
aggAdsA mutagenesis	
K51A	caaaagtaacgacaagtgatcaatgtattgcagccggtgcaaaggt
K55A	aatgtattaaagccggtgcagcggtctggttatggggaac
W57A	gccggtgcaaaggtcgcgttatggggaacagg
W59A	ggtgcaaaggtctggttagcgggaacaggtcccg
K73AK76A	ccgctaataagtgggtcctacagcatgctgcagttgcagcacaaaaatacacattgaatcc
K78A	catacacattgaatccatctatagatggag
I85A	gttgcaaaacaaaaatacacattgaatccatctgcagatggaggtgcagatt
F91A	atctatagatggaggtgcagatgctgtgaaccaaggaactgatg
K103A	gtgaaccaaggaactgatgcaaaaatttataaagcattgacaagcgggaacaa
K109A	ttataaaaaattgacaagcgggaacgcatttctgaacgcaagtgtttcagtc
R152A	acaaacaactcagacaatcgccctcaccgttacataaggg
